# Haem-dependent dimerization of PGRMC1/Sigma-2 receptor facilitates cancer proliferation and chemoresistance

**DOI:** 10.1038/ncomms11030

**Published:** 2016-03-18

**Authors:** Yasuaki Kabe, Takanori Nakane, Ikko Koike, Tatsuya Yamamoto, Yuki Sugiura, Erisa Harada, Kenji Sugase, Tatsuro Shimamura, Mitsuyo Ohmura, Kazumi Muraoka, Ayumi Yamamoto, Takeshi Uchida, So Iwata, Yuki Yamaguchi, Elena Krayukhina, Masanori Noda, Hiroshi Handa, Koichiro Ishimori, Susumu Uchiyama, Takuya Kobayashi, Makoto Suematsu

**Affiliations:** 1 Department of Biochemistry, Keio University School of Medicine, and Japan Science and Technology Agency (JST), Core Research for Evolutional Science and Technology (CREST), Tokyo 160-8582, Japan; 2 Department of Medical Chemistry and Cell Biology, Graduate School of Medicine, Kyoto University, Kyoto 606-8501, Japan; 3 Department of Statistical Genetics, Graduate School of Medicine, Kyoto University, Kyoto 606-8501, Japan; 4 Bioorganic Research Institute, Suntory Foundation for Life Sciences, Kyoto 619-0284, Japan; 5 Department of Chemistry, Faculty of Science, Hokkaido University, Sapporo 060-0810, Japan; 6 JST, Research Acceleration Program, Membrane Protein Crystallography Project, Kyoto 606-8501, Japan; 7 Department of Biological Information, Graduate School of Bioscience and Biotechnology, Tokyo Institute of Technology, Yokohama 226-8501, Japan; 8 Department of Biotechnology, Graduate School of Engineering, Osaka University, Osaka 565-0871, Japan; 9 Department of Nanoparticle Translational Research, Tokyo Medical University, Tokyo 160-8402, Japan; 10 Okazaki Institute for Integrative Bioscience, National Institutes of Natural Sciences, Okazaki 444-8787, Japan; 11 JST, CREST, Kyoto 606-8501, Japan; 12 Platform for Drug Discovery, Informatics, and Structural Life Science, JST, Kyoto 606-8501, Japan; 13 Department of Biochemistry, Keio University School of Medicine, JST, Exploratory Research for Advanced Technology (ERATO), Suematsu Gas Biology Project, Tokyo 160-8582, Japan

## Abstract

Progesterone-receptor membrane component 1 (PGRMC1/Sigma-2 receptor) is a haem-containing protein that interacts with epidermal growth factor receptor (EGFR) and cytochromes P450 to regulate cancer proliferation and chemoresistance; its structural basis remains unknown. Here crystallographic analyses of the PGRMC1 cytosolic domain at 1.95 Å resolution reveal that it forms a stable dimer through stacking interactions of two protruding haem molecules. The haem iron is five-coordinated by Tyr113, and the open surface of the haem mediates dimerization. Carbon monoxide (CO) interferes with PGRMC1 dimerization by binding to the sixth coordination site of the haem. Haem-mediated PGRMC1 dimerization is required for interactions with EGFR and cytochromes P450, cancer proliferation and chemoresistance against anti-cancer drugs; these events are attenuated by either CO or haem deprivation in cancer cells. This study demonstrates protein dimerization via haem–haem stacking, which has not been seen in eukaryotes, and provides insights into its functional significance in cancer.

## Abstract (web summary)

PGRMC1 binds to EGFR and cytochromes P450, and is known to be involved in cancer proliferation and in drug resistance. Here, the authors determine the structure of the cytosolic domain of PGRMC1, which forms a dimer via haem–haem stacking, and propose how this interaction could be involved in its function.

## Graphical abstract

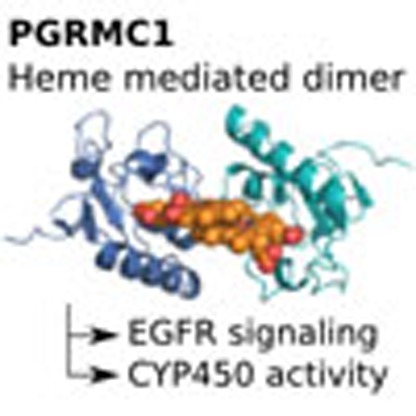

Much attention has been paid to the roles of haem-iron in cancer development. Increased dietary intake of haem is a risk factor for several types of cancer^1^,^2^,^3^. Previous studies showed that deprivation of iron or haem suppresses tumourigenesis^4^,^5^. On the other hand, carbon monoxide (CO), the gaseous mediator generated by oxidative degradation of haem via haem oxygenase (HO), inhibits tumour growth^6^. Thus, a tenuous balance between free haem and CO plays key roles in cancer development and chemoresistance^7^, although the underlying mechanisms are not fully understood.

To gain insight into the underlying mechanisms, we took chemical biological approaches using affinity nanobeads^8^ carrying haem and identified progesterone-receptor membrane component 1 (PGRMC1) as a haem-binding protein from mouse liver extracts (Supplementary Fig. 1). PGRMC1 is a member of the membrane-associated progesterone receptor (MAPR) family^9^ with a cytochrome *b*_5_-like haem-binding region, and is known to be highly expressed in various types of cancers^10^,^11^,^12^,^13^,^14^,^15^,^16^,^17^. PGRMC1 is anchored to the cell membrane through the N-terminal transmembrane helix and interacts with epidermal growth factor receptor (EGFR)^18^ and cytochromes P450 (ref 19). While PGRMC1 is implicated in cell proliferation and cholesterol biosynthesis^20^,^21^, the structural basis on which PGRMC1 exerts its function remains largely unknown.

Here we show that PGRMC1 exhibits a unique haem-dependent dimerization. The dimer binds to EGFR and cytochromes P450 to enhance tumour cell proliferation and chemoresistance. The dimer is dissociated to monomers by physiological levels of CO, suggesting that PGRMC1 serves as a CO-sensitive molecular switch regulating cancer cell proliferation.

## Results

### X-ray crystal structure of PGRMC1

**Figure 1:**
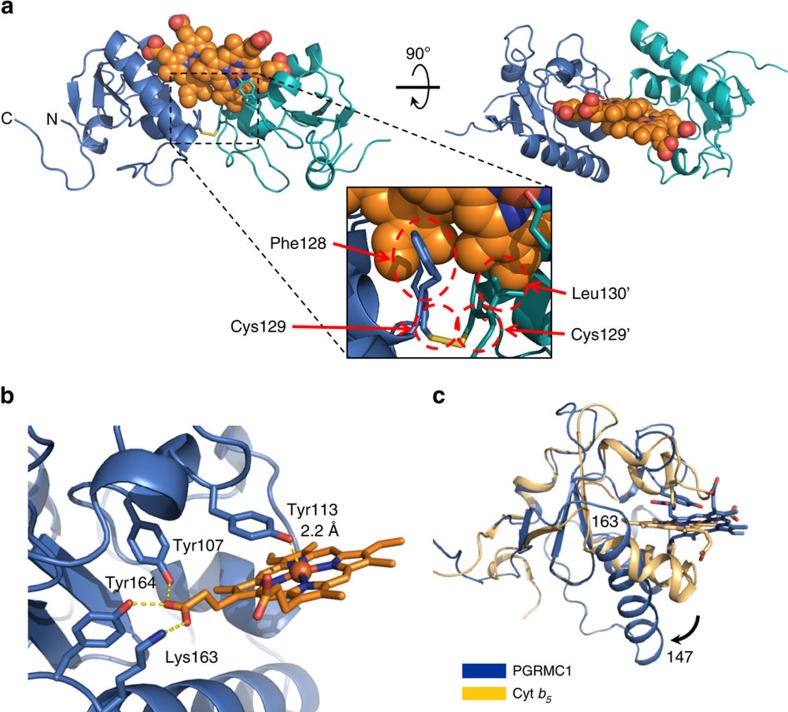
X-ray crystal structure of PGRMC1. (**a**) Structure of the PGRMC1 dimer formed through stacked haems. Two PGRMC1 subunits (blue and green ribbons) dimerize via stacking of the haem molecules. (**b**) Haem coordination of PGRMC1 with Tyr113. Comparison of PGRMC1 (blue) and cytochrome *b*_*5*_ (yellow, ID: 3NER). (**c**) PGRMC1 has a longer helix (a.a.147–163), which is shifted away from the haem (arrow).

We solved the crystal structure of the haem-bound PGRMC1 cytosolic domain (a.a.72–195) at 1.95 Å resolution (Supplementary Fig. 2). In the presence of haem, PGRMC1 forms a dimeric structure largely through hydrophobic interactions between the haem moieties of two monomers (Fig. 1a, Table 1 and Supplementary Fig. 3; a stereo-structural image is shown in Supplementary Fig 4). While the overall fold of PGRMC1 is similar to that of canonical cytochrome *b*_5_, their haem irons are coordinated differently. In cytochrome *b*_5,_ the haem iron is six-coordinated by two axial histidine residues. These histidines are missing in PGRMC1, and the haem iron is five-coordinated by Tyr113 (Y113) alone (Fig. 1b and Supplementary Fig. 3). A homologous helix that holds haem in cytochrome *b*_5_ is longer, shifts away from haem, and does not form a coordinate bond in PGRMC1 (Fig. 1c). Consequently, the five-coordinated haem of PGRMC1 has an open surface that allows its dimerization through hydrophobic haem–haem stacking. Contrary to our finding, Kaluka *et al*.^22^ recently reported that Tyr164 of PGRMC1 is the axial ligand of haem because mutation of this residue impairs haem binding. Our structural data revealed that Tyr164 and a few other residues such as Tyr107 and Lys163 are in fact hydrogen-bonded to haem propionates. This is consistent with observations by Min *et al*.^23^ that Tyr 107 and Tyr113 of PGRMC1 are involved in binding with haem. These amino acid residues are conserved among MAPR family members (Supplementary Fig. 5a), suggesting that these proteins share the ability to exhibit haem-dependent dimerization.

### PGRMC1 exhibits haem-dependent dimerization in solution

In the PGRMC1 crystal, two different types of crystal contacts (chain A–A″ and A–B) were observed in addition to the haem-mediated dimer (chain A–A′) (Supplementary Figs 3 and 6a). To confirm that haem-assisted dimerization of PGRMC1 occurs in solution, we analysed the structure of apo- and haem-bound PGMRC1 by two-dimensional nuclear magnetic resonance (NMR) using heteronuclear single-quantum coherence and transverse relaxation-optimized spectroscopy (Supplementary Figs 6b and 7). NMR signals from some amino acid residues of PGRMC1 disappeared due to the paramagnetic relaxation effect of haem (Supplementary Figs 6b); these residues were located in the haem-binding region. When chemical shifts of apo- and haem-bound forms of PGMRC1 were compared, some amino acid residues close to those which disappeared because of the paramagnetic relaxation effect of haem exhibit notable chemical shifts (Supplementary Fig. 6a,b; dark yellow). However, at the interfaces of the other possible dimeric structures (Supplementary Fig. 6a, chain A–A″; cyan and chain A–B; violet), no significant difference was observed. Furthermore, free energy of dissociation predicted by PISA^24^ suggested that the haem-mediated dimer is stable in solution while the other potential interactions are not. We also attempted to predict the secondary structure of PGRMC1 through NMR data by calculating with TALOS+ program^25^ (Supplementary Fig. 8); the prediction suggested that the overall secondary structure is comparable between apo- and haem-bound forms of PGRMC1 in solution.

**Figure 2:**
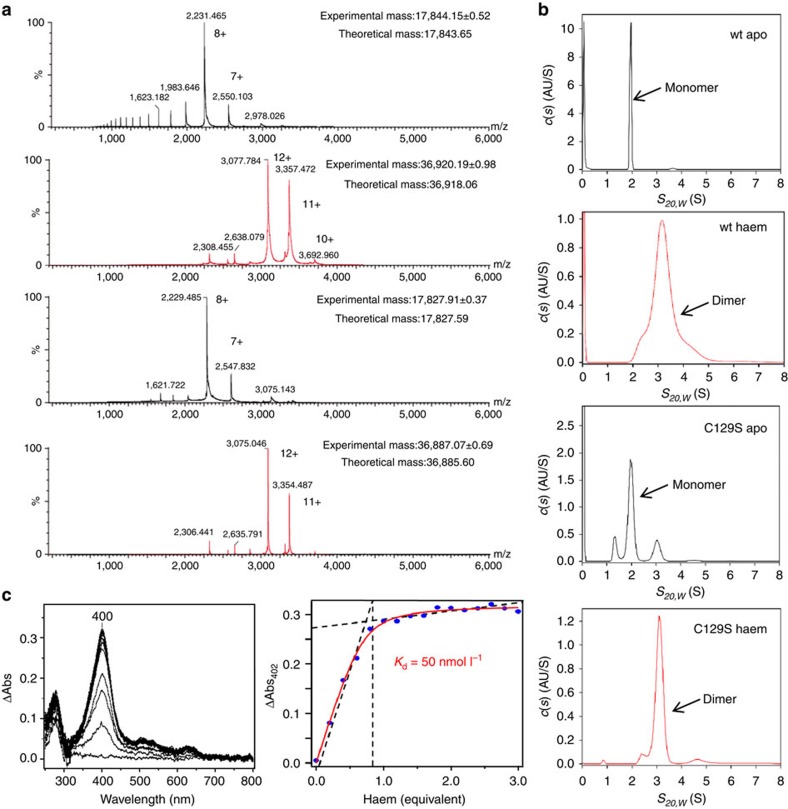
PGRCM1 is dimerized by binding with haem. (**a**) Mass spectrometric analyses of the wild-type (wt) PGRMC1 or the C129S mutant in the presence or absence of haem under non-denaturing condition. Both proteins had identical lengths (a.a.44–195). Data of experimental mass show mean±s.d. (**b**) SV-AUC analyses of the wt-PGRMC1 and the C129S mutant (a.a.44–195) in the presence or absence of haem. SV-AUC experiments were performed with 1.5 mg ml^−1^ of PGRMC1 proteins. The major peak with sedimentation coefficient *S*_20,*w*_ of 1.9∼2.0 S (monomer) or 3.1 S (dimer) was detected. (**c**) Difference absorption spectra of PGRMC1 (a.a.44–195) titrated with haem (left panel). The titration curve of haem to PGRMC1 (right panel). The absorbance difference at 400 nm is plotted against the haem concentration.

We analysed the haem-dependent dimerization of the PGRMC1 cytosolic domain (a.a.44–195) in solution (Fig. 2 and Table 2). Mass spectrometry (MS) analyses under non-denaturing condition demonstrated that the apo-monomer PGRMC1 resulted in dimerization by binding with haem (Fig. 2a). It should be noted that a disulfide bond between two Cys129 residues is observed in the crystal of PGRMC1 (Fig. 1a), while Cys129 is not conserved among the MAPR family proteins (Supplementary Fig. 5a). This observation led us to examine whether or not the disulfide bond contributes to PGRMC1 dimerization. MS analyses under non-denaturing conditions clearly showed that the Cys129Ser (C129S) mutant is dimerized in the presence of haem, indicating that the haem-mediated dimerization of PGRMC1 occurs independently of the disulfide bond formation via Cys129 (Fig. 2a). Supporting this, MS analyses under denaturing conditions showed that haem-mediated PGRMC1 dimer is completely dissociated into monomer, indicating that dimerization of this kind is not mediated by any covalent bond such as disulfide bond (Supplementary Fig. 9).

We also analysed the haem-dependent dimerization of PGRMC1 by diffusion-ordered NMR spectroscopy (DOSY) analyses (Table 2, Supplementary Fig. 10). The results suggested that the hydrodynamic radius of haem-bound PGRMC1 is larger than that of apo-PGRMC1. To further evaluate changes in molecular weights in dimerization of PGRMC1, sedimentation velocity analytical ultracentrifugation (SV-AUC) analysis was carried out. Whereas the wild-type (wt) apo-PGRMC1 appeared at a 1.9 S peak as monomer, the haem-binding PGRMC1 was converted into dimer at a 3.1 S peak (Fig. 2b). Similarly, the C129S mutant of PGRMC1 converted from monomer to dimer by binding to haem (Fig. 2b). SV-AUC analyses also allowed us to examine the stability of haem/PGRMC1 dimer. To this end, we used different concentrations (3.5–147 μmol l^−1^) of haem-bound PGRMC1 protein (a.a. 72–195), which were identical to that used in the crystallographic analysis. The sedimentation coefficients calculated on the basis of the crystal structure were 1.71 S for monomer and 2.56 S for dimer (Supplementary Fig. 11, upper panel). The results showed that the PGRMC1 dimer is not dissociated into monomer at all concentrations examined (Supplementary Fig. 11, lower panel), suggesting that the *K*_*d*_ value of haem-mediated dimer of PGRMC1 is under 3.5 μmol l^−1^. A value of this kind implies that the PGRMC1 dimer is more stable than other dimers of extracellular domain of membrane proteins such as Toll like receptor 9 (dimerization *K*_d_ of 20 μmol l^−1^) (ref. 26) and plexin A_2_ receptor (dimerization *K*_d_ higher than 300 μmol l^−1^) (ref. 27). The current analytical data confirmed that apo-PGRMC1 monomer converts into dimer by binding to haem in solution (Table 2).

We also showed by haem titration experiments that haem binding to PGRMC1 was of low affinity with a *K*_d_ value of 50 nmol l^−1^; this is comparable with that of iron regulatory protein 2, which is known to be regulated by intracellular levels of haem^28^ (Fig. 2c and Supplementary Table 1). These results raised the possibility that the function of PGRMC1 is regulated by intracellular haem concentrations.

### CO inhibits haem-dependent dimerization of PGRMC1

**Figure 3:**
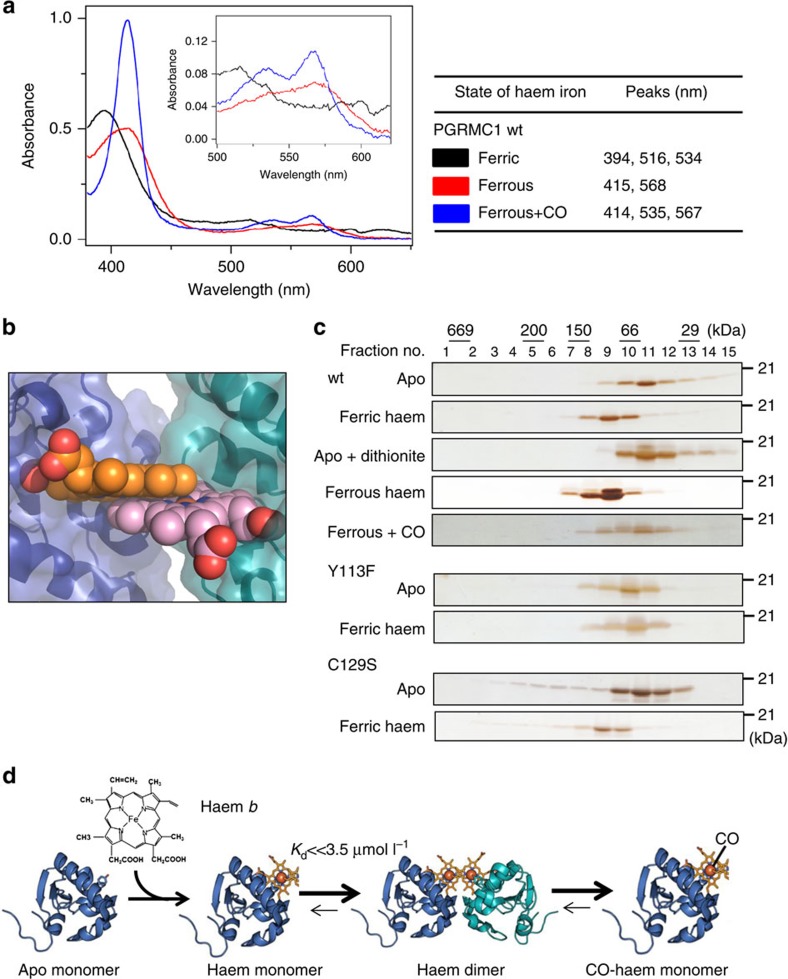
Carbon monoxide inhibits haem-dependent PGRMC1 dimerization. (**a**) UV-visible absorption spectra of PGRMC1 (a.a.44–195). Measurements were performed in the presence of the oxidized form of haem (ferric), the reduced form of haem (ferrous) and the reduced form of haem plus CO gas (ferrous+CO). (**b**) Close-up view of haem stacking. Spheres are drawn with van der Waals radii. (**c**) Gel-filtration chromatography analyses of PGRMC1 (a.a.44–195) wild-type (wt) and the Y113F or C129S mutant in the presence or absence of haem, dithionite and/or CO. (**d**) Transition model for structural regulation of PGRMC1 in response to haem and CO.

Crystallographic analyses revealed that Tyr113 of PGRMC1 is an axial ligand for haem and contributes to haem-dependent dimerization (Fig. 1a). Analysis of UV-visible spectra revealed that the heme of PGRMC1 is reducible from ferric to ferrous state, thus allowing CO binding (Fig. 3a). Furthermore, the UV-visible spectrum of the wild type PGRMC1 was the same as that of the C129S mutant of PGRMC1, and the *R*/*Z* ratio determined by the intensities between the Soret band (394 nm) peak and the 274-nm peak showed that these proteins were fully loaded with haem (Supplementary Fig. 12). Analysis of the ferric form of PGRMC1 using resonance Raman spectroscopy (Supplementary Fig. 13) showed that the relative intensity of oxidation and spin state marker bands (*ν*_4_ and *ν*_3_) is close to 1.0, which is consistent with it being a haem protein with a proximal Tyr coordination^29^. A specific Raman shift peaking at *v*_Fe–CO_=500 cm^−1^ demonstrated that the CO-bound haem of PGRMC1 is six-coordinated (Supplementary Fig. 13).

Since PGRMC1 dimerization involves the open surface of haem on the opposite side of the axial Tyr113, no space for CO binding is available in the dimeric structure (Fig. 3b). This prompted us to ask if CO binding to haem causes dissociation of the PGRMC1 dimer. Analysis by gel filtration chromatography revealed that the relative molecular sizes of the wild-type and the C129S mutant of PGRMC1 are increased by adding haem to apo-PGRMC1 regardless of the oxidation state of the iron (Fig. 3c), which is in agreement with the results in Table 1. CO application to ferrous PGRMC1 abolished the haem-dependent increase in its molecular size. Under this reducing condition in the presence of dithionite, analyses of UV-visible spectra indicated that CO-binding with haem-PGRMC1 is stable, showing only 20% reduction of the absorbance at 412 nm within 2 h (Supplementary Fig. 14). Furthermore, the Tyr113Phe (Y113F) mutant of PGRMC1 was not responsive to haem. These results suggest that CO favours the six-coordinate form of haem and interferes with the haem-mediated dimerization of PGRMC1. To examine the inhibitory effects of CO on haem-mediated PGRMC1 dimerization, SV-AUC analysis was carried out. The peak corresponding to the haem/PGRMC1 dimer was detected under reducing conditions in the presence of dithionite (Supplementary Fig. 15, middle panel). Under these circumstances, CO application induced dissociation of the haem-mediated dimers of PGRMC1 to generate a peak of monomers (Supplementary Fig. 15, lower panel). These observations raised the transition model for structural regulation of PGRMC1 in response to haem (Fig. 3d). As mentioned above, apo-PGRMC1 exists as monomer. By binding with haem (binding *K*_d_=50 nmol l^−1^), PGRMC1 forms a stable dimer (dimerization *K*_d_<<3.5 μmol l^−1^) through stacking of the two open surfaces of the five-coordinated haem molecules in each monomer. CO induces the dissociation of the haem-mediated dimer of PGRMC1 by interfering with the haem-stacking interface via formation of the six-coordinated CO-haem-PGRMC1 complex. Such a dynamic structural regulation led us to further examine the regulation of PGRMC1 functions in cancer cells.

### PGRMC1 dimerization is required for binding to EGFR

**Figure 4:**
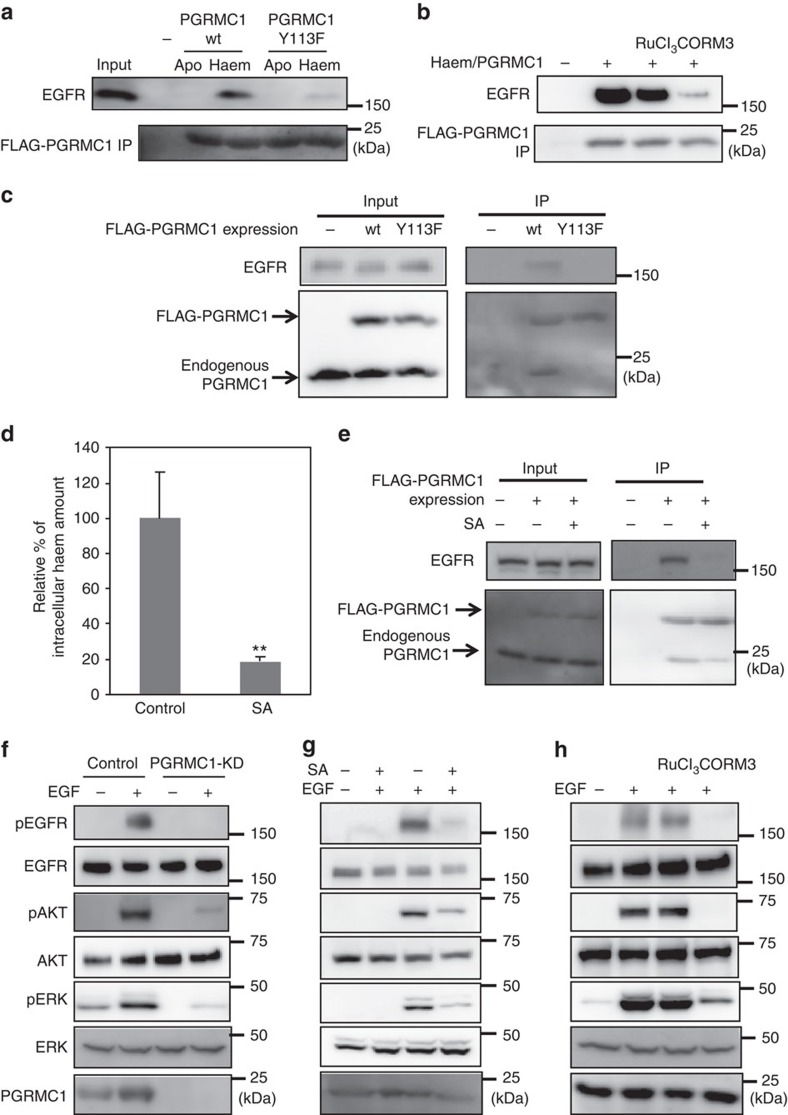
Haem-dependent dimerization of PGRMC1 is necessary for tumour proliferation mediated by EGFR signalling. (**a**) FLAG-PGRMC1 wild-type (wt) and Y113F mutant proteins (a.a.44–195), in either apo- or haem-bound form, were incubated with purified EGFR and co-immunoprecipitated with anti-FLAG antibody-conjugated beads. Input and bound proteins were detected by Western blotting. (**b**) *In vitro* binding assay was performed as in (**a**) using haem-bound FLAG-PGRMC1 wt (a.a.44–195) and purified EGFR with or without treatment of RuCl_3_ and CORM3. (**c**) FLAG-PGRMC1 wt or Y113F (full length) was over-expressed in HCT116 cells and immunoprecipitated with anti-FLAG antibody-conjugated beads. Co-immunoprecipitated proteins (FLAG-PGRMC1, endogenous PGRMC1 and EGFR) were detected with Western blotting by using anti-PGRMC1 or anti-EGFR antibody. (**d**) HCT116 cells were treated with or without 250 μmol l^−1^ of succinylacetone (SA) for 48 h. The intracellular haem was extracted and quantified by reverse-phase HPLC. The data represent mean±s.d. of four separate experiments. **P<0.01 using unpaired Student's *t*-test. (**e**) Co-immunoprecipitation assay was performed as in (**c**) with or without SA treatment in HCT116 cells. (**f**) HCT116 cells expressing control shRNA or those knocking down PGRMC1 (PGRMC1-KD) were treated with EGF or left untreated, and components of the EGFR signaling pathway were detected by Western blotting. (**g**,**h**) HCT116 cells were treated with or without EGF, SA, RuCl_3_ and CORM3 as indicated, and components of the EGFR signaling pathway were detected by Western blotting.

Because PGRMC1 is known to interact with EGFR and to accelerate tumour progression^18^, we examined the effect of haem-dependent dimerization of PGRMC1 on its interaction with EGFR by using purified proteins. As shown in Fig. 4a, the cytosolic domain of wild-type PGRMC1, but not the Y113F mutant, interacted with purified EGFR in a haem-dependent manner. This interaction was disrupted by the ruthenium-based CO-releasing molecule, CORM3, but not by RuCl_3_ as a control reagent (Fig. 4b). We further analysed the intracellular interaction between PGRMC1 and EGFR. FLAG-tagged PGRMC1 ectopically expressed in human colon cancer HCT116 cells was immunoprecipitated with anti-FLAG antibody, and co-immunoprecipitated EGFR and endogenous PGRMC1 binding to FLAG-PGRMC1 were detected by Western blotting (Fig. 4c). The C129S mutant of PGRMC1 also interacted with endogenous PGRMC1 and EGFR (Supplementary Fig. 16). Whereas FLAG-tagged wild-type PGRMC1 interacted with endogenous PGRMC1 and EGFR, the Y113F mutant did not. We also examined the effect of succinylacetone (SA), an inhibitor of haem biosynthesis (Fig. 4d). As expected, SA significantly reduced PGRMC1 dimerization and its interaction with EGFR (Fig. 4e), indicating that haem-mediated dimerization of PGMRC1 is critical for its binding to EGFR.

### PGRMC1 dimer facilitates EGFR-mediated cancer growth

Next, we investigated the functional significance of PGRMC1 dimerization in EGFR signaling. EGF-induced phosphorylations of EGFR and its downstream targets AKT and ERK were decreased by PGRMC1 knockdown (PGRMC1-KD) (Fig. 4f). Similarly, EGFR signaling was suppressed by treatment of HCT116 cells with SA (Fig. 4g) or CORM3 (Fig. 4h). These results suggested that haem-mediated dimerization of PGRMC1 is critical for EGFR signaling.

**Figure 5:**
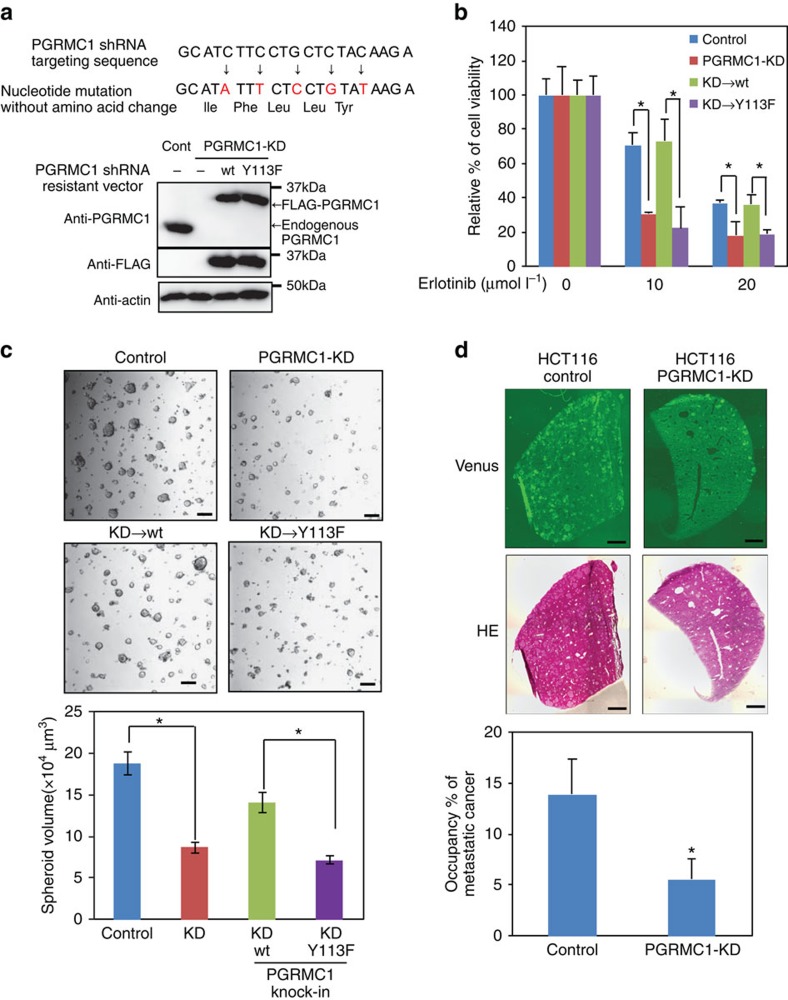
Haem-dependent dimerization of PGRMC1 accelerates tumour growth through the EGFR signaling pathway. (**a**) Nucleotide sequences of PGRMC1 targeted by shRNA and of the shRNA-resistant full length PGRMC1 expression vector. Stable PGRMC1-knockdown (PGRMC1-KD) HCT116 cells were transiently transfected with the shRNA-resistant expression vector of wild-type PGRMC1 (wt) or the Y113F mutant (Y113F). (**b**) Erlotinib was added to HCT116 (control) cells, PGRMC1-KD cells or PGRMC1-KD cells expressing shRNA-resistant PGRMC1 wt or Y113F, and cell viability was examined by MTT assay. The data represent mean±s.d. of four separate experiments. *P<0.01 using ANOVA with Fischer's LSD test. (**c**) Spheroid formation in control and PGRMC1-KD HCT116 cells. Pictures indicate representative micrographs of spheroids formed under each condition. Spheroid size were measured and compared among groups. The graph represents mean±s.e. of each spheroid size. *P<0.01 using ANOVA with Fischer's LSD test. Scale bar: 0.1 mm. (**d**) Tumour-bearing livers of NOG mice at 10 days after intrasplenic injection of HCT116 (control) or PGRMC1-KD cells. Percentages of cross-sectional areas showing metastatic tumours were calculated. Data represent mean±s.d. of 10 separate experiments. *P<0.05 using unpaired Student's *t*-test. Scale bar: 5 mm.

To further investigate the role of the dimerized form of PGRMC1 in cancer proliferation, we performed PGRMC1 knockdown-rescue experiments using FLAG-tagged wild-type and Y113F PGRMC1 expression vectors, in which silent mutations were introduced into the nucleotide sequence targeted by shRNA (Fig. 5a). While proliferation of HCT116 cells was not affected by knocking down PGRMC1, PGRMC1-KD cells were more sensitive to the EGFR inhibitor erlotinib than control HCT116 cells, and the knockdown effect was reversed by co-expression of shRNA-resistant wild-type PGRMC1 but not of the Y113F mutant (Fig. 5b). Chemosensitivity enhancement by two different shRNAs to PGRMC1 was seen also in HCT116 cells and human hepatoma HuH7 cells (Supplementary Fig. 17). Furthermore, PGRMC1-KD inhibited spheroid formation of HCT116 cells in culture, and this inhibition was reversed by co-expression of wild-type PGRMC1 but not of the Y113F mutant (Fig. 5c and Supplementary Fig. 18). Thus, PGRMC1 dimerization is important for cancer cell proliferation and chemoresistance.

We examined the role of PGRMC1 in metastatic progression by xenograft transplantation assays using super-immunodeficient NOD/scid/γnull (NOG) mice^7^,^30^,^31^. Ten days after intra-splenic implantation of HCT116 cells that were genetically tagged with a fluorescent protein Venus, the group implanted with PGRMC1-KD cells showed a significant decrease of liver metastasis in comparison with the control group (Fig. 5d).

### Interaction of PGRMC1 dimer with cytochromes P450

**Figure 6:**
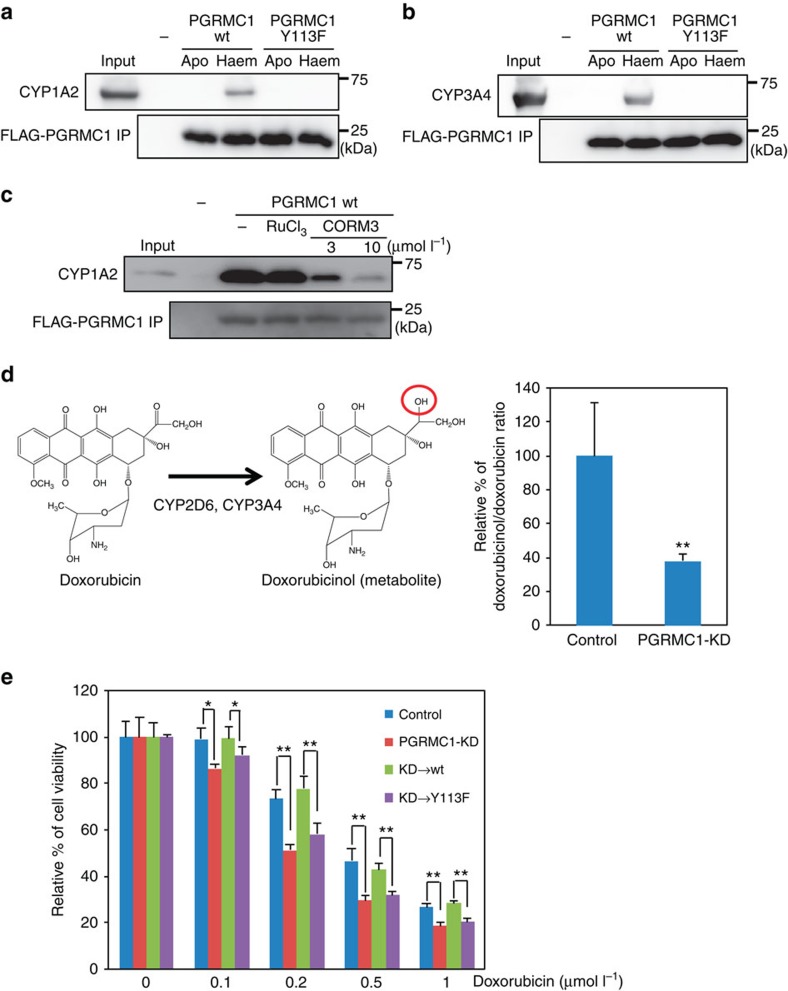
Haem-dependent PGRMC1 dimerization enhances tumour chemoresistance through interaction with cytochromes P450. (**a**,**b**) FLAG-PGRMC1 wild-type (wt) and Y113F mutant proteins (a.a.44–195), in either apo or haem-bound form, were incubated with CYP1A2 (**a**) or CYP3A4 (**b**) and immunoprecipitated with anti-FLAG antibody-conjugated beads. Input and bound proteins were detected by Western blotting. (**c**) Binding assay was performed as in (**a**) using haem-bound FLAG-PGRMC1 wt and CYP1A2 with or without RuCl_3_ and CORM3. (**d**) Schematic illustration of doxorubicin metabolism is shown on the left. Doxorubicin was incubated with HCT116 cells expressing control shRNA or shPGRMC1 (PGRMC1-KD), and the doxorubicinol/doxorubicin ratios in cell pellets were determined using LC-MS. Data represent mean±s.d. of four separate experiments. **P<0.01 versus control using unpaired Student's *t*-test. (**e**) Indicated amounts of doxorubicin were added to HCT116 (control) cells, PGRMC1-KD cells, or PGRMC1-KD cells expressing shRNA-resistant full-length PGRMC1 wt or Y113F, and cell viability was examined by MTT assay. Data represent mean±s.d. of four separate experiments. *P<0.01 using ANOVA with Fischer's LSD test.

Since PGRMC1 has been shown to interact with cytochromes P450 (ref 19), we investigated whether the haem-mediated dimerization of PGRMC1 is necessary for their interactions. Recombinant CYP1A2 and CYP3A4 including a microsomal formulation containing cytochrome *b*_5_ and cytochrome P450 reductase, drug-metabolizing cytochromes P450, interacted with wild-type PGRMC1, but not with the Y113F mutant, in a haem-dependent manner (Fig. 6a,b). Moreover, the interaction of PGRMC1 with CYP1A2 was blocked by CORM3 under reducing conditions (Fig. 6c), indicating that PGRMC1 dimerization is necessary for its interaction with cytochromes P450. Doxorubicin is an anti-cancer reagent that is metabolized into inactive doxorubicinol by CYP2D6 and CYP3A4 (Fig. 6d)^32^,^33^. PGRMC1-KD significantly suppressed the conversion of doxorubicin to doxorubicinol (Fig. 6d) and increased sensitivity to doxorubicin (Fig. 6e). Enhanced doxorubicin sensitivity was modestly but significantly induced by PGRMC1-KD. This effect was reversed by co-expression of the wild-type PGRMC1 but not of the Y113F mutant, suggesting that PGRMC1 enhances doxorubicin resistance of cancer cells by facilitating its degradation via cytochromes P450. To gain further insight into the interaction between PGRMC1 and cytochromes P450, surface plasmon resonance analyses were conducted using recombinant CYP51 and PGRMC1. This was based on a previous study showing that PGRMC1 binds to CYP51 and enhances cholesterol biosynthesis by CYP51 (refs 19, 34). CYP51 interacted with PGRMC1 in a concentration-dependent manner in the presence of haem, but not in its absence (Supplementary Fig. 19), suggesting the requirement for the haem-dependent dimerization of PGRMC1. The *K*_d_ value of PGRMC1 binding to CYP51 was in a micromolar range and comparable with those of other haem proteins, such as cytochrome P450 reductase^35^ and neuroglobin/*Gα*_*i1*_ (ref. 36), suggesting that haem-dependent PGRMC1 interaction with CYP51 is biologically relevant.

## Discussion

In this study, we showed that PGRMC1 dimerizes by stacking interactions of haem molecules from each monomer. Recently, Lucas *et al*.^37^ reported that translationally-controlled tumour protein was dimerized by binding with haem, but its structural basis remains unclear. This is the report showing crystallographic evidence that indicates roles of the direct haem–haem stacking in haem-mediated dimerization in eukaryotes, although a few examples are known in bacteria^38^. Sequence alignments show that haem-binding residues (Tyr113, Tyr107, Lys163 and Tyr164) in PGRMC1 are conserved among MAPR proteins (Supplementary Fig. 5). In the current study, the Y113 residue plays a crucial role for the haem-dependent dimerization of PGRMC1 and resultant regulation of cancer proliferation and chemoresistance (Figs 5c and 6e). Since the Y113 residue is involved in the putative consensus motif of phosphorylation by tyrosine kinases such as Abl and Lck^39^, we investigated whether phosphorylated Y113 is present in HCT116 cells by ESI-MS analysis. It was, however, undetectable under current experimental conditions (Supplementary Fig. 20). Recently, Peluso *et al*.^40^ reported that PGRMC1 binds to PGRMC2, suggesting that MAPR family members may also undergo haem-mediated heterodimerization.

We showed that the haem-mediated dimer of PGRMC1 enables interaction with different subclasses of cytochromes P450 (CYP) (Fig. 6). While the effects of PGRMC1 on cholesterol synthesis mediated by CYP51 have been well documented in yeast^19^,^41^ and human cells^34^, it has not been clear whether drug-metabolizing CYP activities are regulated by PGRMC1. Szczesna-Skorupa and Kemper^34^ reported that PGRMC1 exhibited an inhibitory effect on CYP3A4 drug metabolizing activity by competitively binding with cytochrome P450 reductase (CPR) in HEK293 or HepG2 cells. On the other hand, Oda *et al*.^42^ reported that PGRMC1 had no effect to CYP2E1 and CYP3A4 activities in HepG2 cell. Several other groups showed that PGRMC1 enhanced chemoresistance in several cancer cells such as uterine sarcoma^43^, breast cancer^17^, endometrial tumour^13^ and ovarian cancer^44^,^45^; however, no evidence of PGRMC1-dependent regulation of CYP activity was provided. Our results showed that PGRMC1 contributes to enhancement of the doxorubicin metabolism, which is mediated by CYP2D6 or CYP3A4 in human colon cancer HCT116 cells (Fig. 6d). While the effects of structural diversity of CYP family proteins and interactions with different xenobiotic substrates should further be examined, the current results suggest that the interaction of drug-metabolizing CYPs with the haem-mediated dimer of PGRMC1 plays a crucial role in regulating their activities.

**Figure 7:**
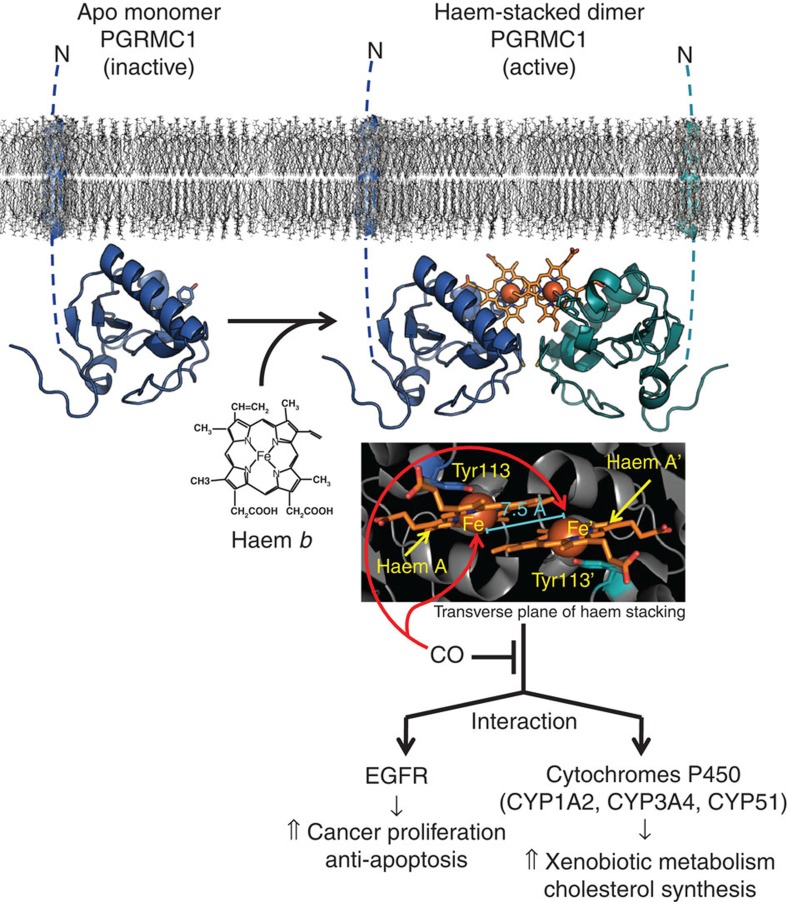
Schematic diagram for the regulation of PGRMC1 functions. Apo-PGRMC1 exists as an inactive monomer. On binding to haem, PGRMC1 forms a dimer through stacking interactions between the haem moieties, which enables PGRMC1 to interact with EGFR and cytochromes P450, leading to an enhanced proliferation and chemoresistance of cancer cells. CO interferes with the stacking interactions of the haems and thereby inhibits PGRMC1 functions.

We showed that haem-mediated dimerization of PGRMC1 enhances proliferation and chemoresistance of cancer cells through binding to and regulating EGFR and cytochromes P450 (illustrated in Fig. 7). Since the haem-binding affinity of PGRMC1 is lower than those of constitutive haem-binding proteins such as myoglobin, PGMRC1 is probably interconverted between apo-monomer and haem-bound dimer forms in response to changes in the intracellular haem concentration. Considering microenvironments in and around malignant tumours, the haem concentration in cancer cells is likely to be elevated through multiple mechanisms, such as (i) an increased intake of haem, (ii) mutation of enzymes in TCA cycle (for example, fumarate hydratase) that increases the level of succinyl CoA, a substrate for haem biosynthesis and (iii) metastasis to haem-rich organs such as liver, brain and bone marrow^46^,^47^,^48^. Moreover, exposure of cancer cells to stimuli such as hypoxia, radiation and chemotherapy causes cell damages and leads to protein degradation, resulting in increased levels of TCA cycle intermediates and in an enhanced haem biosynthesis^49^,^50^. On the other hand, excessive haem induces HO-1, the enzyme that oxidatively degrades haem and generates CO. Thus, HO-1 induction in cancer cells may inhibit the haem-mediated dimerization of PGRMC1 through the production of CO and thereby suppress tumour progression. This idea is consistent with the observation that HO-1 induction or CO inhibits tumour growth^6^,^51^.

Besides the regulatory roles of PGRMC1/Sigma-2 receptor in proliferation and chemoresistance in cancer cells (ref. 52), recent reports show that PGRMC1 is able to bind to amyloid beta oligomer^53^ to enhance its neurotoxicity^53^,^54^. Furthermore, Sigma-2 ligand-binding is decreased in transgenic amyloid beta deposition model APP/PS1 female mice^55^. These results suggest a possible involvement of PGRMC1 in Alzheimer's disease. The roles of haem-dependent dimerization of PGRMC1 in the functional regulation of its target proteins deserve further studies to find evidence that therapeutic interventions to interfere with the function of the dimer may control varied disease conditions.

## Methods

### Materials

Recombinant EGF, CYP1A2 and CYP3A4 proteins were purchased from Sigma. Erlotinib was purchased from Cayman. Doxorubicin was purchased from Wako. Anti-FLAG (M2) antibody, FLAG peptide and anti-FLAG antibody-conjugated agarose were purchased from Sigma. Haemin and protoporphyrin-IX (PP-IX) were purchased from Porphyrin Science.

### Plasmid constructions

Human PGRMC1 cDNA was cloned from the cDNA library of HuH7 cells. The PGMRC1 (a.a.44–195 for *in vitro* studies and a.a.72–195 for crystallographic analyses and SV-AUC) cDNA fragment was amplified with PCR, digested with Bam HI and Sal I and then ligated into pGEX6P-1 (GE Healthcare). For NMR analysis, the PGRMC1 (a.a.44–195) cDNA fragment was amplified with PCR (with primers containing the factor Xa site) and ligated into the Bam HI and Sal I sites of pGEX6P-1. The full-length PGRMC1 cDNA fragment containing resistant sequences for shRNA was generated by using the primers (Supplementary Methods), and ligated into the Eco RI and Bam HI sites of the C-terminus of the 3xFLAG-tagged expression vector p3xFLAG CMV14 (Sigma).

### Preparations of recombinant proteins

pGEX-PGRMC1 wt, Y113F or C129S mutant expression vectors were transformed into BL21 (DE3), and the bacteria were incubated in LB with ampicillin at 37 °C until OD600 reached at 0.8. Protein expression was induced by 1 mmol l^−1^ isopropyl-β-thiogalactopyranoside for 4 h at 37 °C. Cell pellets were resuspended in the buffer containing 20 mmol l^−1^ Tris-HCl (pH 7.5), 100 mmol l^−1^ NaCl and 0.1% Tween 20, sonicated twice for 5 min at 4 °C and centrifuged at 20,000 × *g* for 30 min. The supernatant was incubated with glutathione Sepharose 4B (GE Healthcare) for 1 h at 4 °C. The resin was then washed five times with the same buffer, and the GST tag was cleared by addition of Precision Protease (GE Healthcare) and further incubation for 16 h at 4 °C. The apo-PGRMC1 was prepared by eliminating the bacterial holo-PGRMC1 with size-exclusion chromatography (Superdex 200; GE Healthcare). Haem-bound PGRMC1 were prepared by treatment with 100 μmol l^−1^ haemin and purified by size-exclusion chromatography. The PGRMC1 protein treated with Precision Protease to cleave the GST-tag contained additional amino acid residues (GPLGSEF) derived from the restriction site and the protease site for Precision Protease at the N-terminal region of PGRMC1.

Isotope-labelled PGRMC1 proteins for NMR analyses were prepared by growing cells (BL21 (DE3)) in minimal M9 media in H_2_O or 99.9% ^2^H_2_O, including ampicillin, metals, vitamins, ^15^N-ammonium chloride and ^13^C or ^12^C glucose as sources of nitrogen and carbon, respectively. These procedures were followed by addition of 1 mmol l^−1^ isopropyl-β-thiogalactopyranoside for 40 h at 20 °C. Protein purification was performed as mentioned above. The GST tag was cleaved with Factor Xa (GE Healthcare). The proteins were treated with Factor Xa to cleave the GST tag at the direct site of N-terminal region of PGRCM1 (a.a.44–195).

### X-ray crystallography

PGRMC1 (a.a.72–195) crystals were grown at 20 °C using hanging-drop vapour diffusion by mixing equal volumes of protein solution and reservoir solution containing 100 mmol l^−1^ sodium cacodylate (pH 6.5) and 1.26–1.45 mol l^−1^ ammonium sulphate. Brown crystals reached maximum size in three weeks. The crystals were soaked in reservoir solution containing 30% trehalose and then flash-frozen in liquid nitrogen. The X-ray diffraction data for PGRMC1 crystals were collected at SPring-8 BL41XU and processed with XDS^56^. The initial phase was obtained by single-wavelength anomalous dispersion, using a dataset collected at 1.73 Å with PHENIX AutoSol^57^. Manual modeling and refinement were performed with COOT^58^ and phenix.refine^59^. The deposited model was refined to a resolution of 1.95 Å. In this model, 95.4% of the residues were in favoured regions of Ramachandran plot, and all the others were in allowed regions. Data collection and refinement statistics are shown in Table 1. Molecular figures were created by PyMOL (Schrodinger, LLC. The PyMOL Molecular Graphics System, Version 1.5.0.3).

### Mass spectrometry analyses

The purified PGRMC1 (a.a.44–195) proteins, the wild-type (apo and haem) and the C129S mutant (apo and haem), which included additional amino acid residues (GPLGSEF), were buffer-exchanged into 100 mmol l^−1^ ammonium acetate, pH 7.5, by passing the proteins through a Bio-Spin 6 column (Bio-Rad). The buffer-exchanged PGRMC1 wild-type (apo and haem) and PGMRC1 C129S mutants were immediately analysed by nanoflow electrospray ionization MS using gold-coated glass capillaries made in house. In the case of ESI-MS analyses under denaturing conditions, buffer-exchanged proteins were denatured before ESI-MS analyses by adding aliquots of formic acid at final concentration of 30%). Spectra were recorded on a SYNAPT G2 HDMS mass spectrometer (Waters, Manchester, UK) in positive ionization mode at 1.20 kV with a 120 V sampling cone voltage. The spectra were calibrated using 1 mg ml^−1^ caesium iodide and analysed with Mass Lynx software (Waters).

### SV-AUC analyses

SV-AUC experiments were performed in a ProteomeLab XL-I analytical ultracentrifuge (Beckman Coulter) equipped with 4-hole An60Ti rotors at 20 °C using Beckman Coulter 12-mm double-sector aluminium centerpieces and sapphire windows. Recombinant PGRMC1 proteins were diluted with the buffer (20 mmol l^−1^ Tris-HCl (pH 7.5) and 100 mmol l^−1^ NaCl) at the indicated concentration. Scanning was performed as quickly as possible at 262,080 *g* at 6.5 cm (60,000 rpm), between 6.0 and 7.2 cm from the axis of rotation with a radial increment of 30 *μ*m using an absorbance optical system. The sedimentation coefficient distributions were obtained using the *c(s)* method of SEDFIT^60^. The partial specific volume, buffer density and viscosity were calculated using the program SEDNTERP 1.09 and were 0.7216, cm^3^ g^−1^, 1.00293, g ml^−1^, and 1.017 cP, respectively. The sedimentation coefficient of PGRMC1 (a.a.72–195) was calculated with the UltraScan Solution Modeler (US-SOMO) suite^61^ using the crystal structure determined in this study. To analyse the effect of CO, protein samples were prepared in a deaerated solution and treated with dithionite at 5 mmol l^−1^ and/or CO gas.

### DOSY analysis

Diffusion-ordered 2D NMR spectroscopy (DOSY) was used to investigate the oligomerization state of PGRMC1 (a.a.44–195) induced by haem binding. Apo- or haem-bound PGRMC1 and reference proteins (including hen egg lysozyme, ovalbumin and bovine serum albumin (BSA)) dissolved in 50 mmol l^−1^ phosphate buffer (pH 7.0) containing 5% D_2_O were measured at 25 °C. The protein concentrations were 0.15–0.2 mmol l^−1^. DOSY spectra were measured using the stimulated echo sequence with a longitudinal-eddy-current delay^62^,^63^, and diffusion coefficients were calculated from signals in the aliphatic regions using the software TOPSPIN (Bruker). The signal intensities fit the Stejskal-Tanner equation:


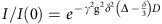


where *I* represents the signal intensity when gradient pulses of length *δ* are applied at strength *g*, varying from 2 to 95% of the full gradient strength (55 G cm^−1^). The diffusion coefficient *D*_25_ is estimated by curve fitting with the term *I*(0), which corresponds to the signal intensity at a gradient strength of 0. The term *γ* represents the gyromagnetic ratio, and Δ represents the delay between two sets of gradients responsible for the stimulated echo. In this study, *δ* was set to 8 or 10 ms, and Δ was set to 40 ms. The hydrodynamic radii of the proteins were estimated on the basis of the Stokes–Einstein equation as follows:


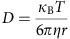


where *T* is the absolute temperature, *r* is the hydrodynamic radius of the spherical molecule, *η* is the viscosity of the solvent, and *κ*_B_ is the Boltzmann constant. The molecular weights (MWs) of apo- and haem-bound PGRMC1 proteins were estimated from a relationship between *r* and MWs. MWs of apo and haem-bound PGRMC1 proteins were obtained from the linear-fitting of measured *r* values for the reference proteins with known MWs according to the following equation: MW=1.2864 (*r*^3^)+8.0411.

### UV-visible absorption spectrometry and haem titration analysis

UV-visible absorption spectra of the protein were recorded with a V-660 (Jasco) spectrophotometer at room temperature. Haem binding was tracked by difference spectroscopy in the Soret region of the UV-visible spectrum. Successive aliquots of 0.5 mmol l^−1^ haemin in *N*,*N*-dimethylformamide were added to both the sample cuvette, which contained 10 μmol l^−1^ apo-PGRMC1 (a.a.44–195), and the reference cuvette. Spectra were recorded 3 min after the addition of each haem aliquot. The absorbance difference at 400 nm was plotted as a function of haem concentration, and the dissociation constant (*K*_d_) was calculated using a quadratic binding equation.

### Gel filtration chromatography

Recombinant PGRMC1 (a.a.44–195) (10 μg) wt, Y113F or C129S mutant, treated with 5 mmol l^−1^ sodium dithionite and/or CO gas or left untreated, was separated on a Superdex 200 column equilibrated in buffer containing 20 mmol l^−1^ Tris-HCl (pH 7.5) and 100 mmol l^−1^ NaCl using a SMART system (GE Healthcare). To prepare the reducing conditions for ferrous haem proteins, the running buffer was deaerated by boiling and saturating it with argon gas according to modified versions of previously reported methods^64^,^65^. Namely, immediately after adding dithionite to give a final concentration of 5 mmol l^−1^, the buffer was equilibrated into the column. The SMART system was sealed with gas-tight taping to maintain anaerobic conditions. Separations of proteins were completed within 1 h. Protein samples were also prepared in the deaerated solution and treated with dithionite at 5 mmol l^−1^ and/or CO gas, right before being injected into the column. Fractions were then subjected to SDS-PAGE under ambient conditions and visualized by silver staining. The size of proteins was estimated using molecular mass markers (thyroglobin, 669 kDa; catalase, 232 kDa; aldolase, 150 kDa; bovine albumin, 66 kDa and β-amylase, 20 kDa). Results showing that the molecular size of PGRMC1 became smaller in CO-treated conditions (Fig. 3) were collected ∼60 min after the start of experiments. The stability of CO-binding to PGRMC1 was examined with UV-visible absorption spectra to chase temporal alterations for 2 h, as shown in Supplementary Fig. 14.

### *In vitro* binding assays

For *in vitro* binding assays, EGFR protein was obtained from ENZO (BML-SE116) as full length protein isolated from human A431 cells. Human CYP1A2, CYP3A4 proteins purified as a microsomal formulation containing cytochrome *b*_*5*_ and cytochrome P450 reductase were obtained from Sigma (C1561 and C4982, respectively). Proteins for human CYP1A2, CYP3A4 or EGFR (1 μg) were incubated with 10 μg of FLAG-PGRMC1 (a.a.44–195) treated with or without 50 μmol l^−1^ haemin in 500 μl of binding buffer containing 20 mmol l^−1^ HEPES-NaOH (pH 7.9), 100 mmol l^−1^ NaCl, 0.2 mmol l^−1^ EDTA, 10% glycerol and 0.1% NP40 for 60 min at room temperature. 5 mmol l^−1^ sodium dithionite was added to produce the reducing conditions specified in the aforementioned methods, and the effects of CORM3 or RuCl_3_ at 10 μmol l^−1^ were examined. Then, 10 μl of equilibrated anti-FLAG (M2) agarose was added to the mixture, which was then incubated for 60 min at room temperature. Bound proteins were washed three times with 200 μl of binding buffer and eluted with 10 μl of 2 μg ml^−1^ FLAG peptide. The eluates were subjected to SDS-PAGE and visualized by Western blotting using antibodies against CYP1A2, CYP3A4 (Santa Cruz: sc-30085 and sc-53850, respectively), FLAG and EGFR (Cell signaling: #2232S).

### Cell culture analyses

The human colon cancer cell line HCT116 and human hepatoma cell line HuH7 were maintained in DMEM medium containing 10% FCS. To generate a stable PGRMC1 knockdown cell line, lentivirus vectors encoding a control or PGRMC1 targeting shRNA sequence were transfected into 293FT cells. The lentivirus was prepared according to the manufacturer's instructions (Invitrogen). HCT116 and HuH7 cells were infected with the lentivirus, and a stable cell line was selected by maintaining the cells in medium containing 10 μg ml^−1^ blasticidin (Invitrogen) for 1 week.

For co-immunoprecipitation assay, the expression vector of FLAG-PGRMC1 or an empty vector into HCT116 by using a transfection reagent Lipofectamine 2000 (Invitrogen). Cells were incubated with or without 250 μ mol l^−1^ succinylacetone (SA) for 48 h, and the cells were then lysed with NP40 lysis buffer (20 mmol l^−1^ Tris-HCl (pH 7.5), 150 mmol l^−1^ NaCl, 1% NP40). The lysates were incubated with 10 μl of equilibrated anti-FLAG (M2) agarose for 60 min at room temperature. Bound proteins were washed three times, and were subjected to SDS-PAGE and visualized by Western blotting using antibodies against PGRMC1 (NOVUS: NBP1–83220) and EGFR.

For analysis of EGFR signaling, cells were incubated overnight with serum-deprived medium, and then 100 ng ml^−1^ EGF was added for 5 min. Cells were lysed with RIPA buffer, and the lysates were subjected to SDS-PAGE and visualized by Western blotting using antibodies against PGRMC1, EGFR, phospho-Y1068 EGFR (Cell signaling: #2234S), AKT (Cell signaling: #9272S), phospho-S473AKT (Cell signaling: #4060S), ERK (Cell signaling: #4695S) and phospho-T185 Y187 ERK (Invitrogen: 44680 G).

To analyse proliferation of HCT116 cells, Lipofectamine 2000 (Invitrogen) was used to transfect the shRNA-resistant expression vector of FLAG-PGRMC1 or an empty vector into HCT116 control or PGRMC1-knockdown cells. After 24 h, the cells were seeded and incubated for 12 h on a 96-well plate, after which erlotinib or doxorubicin was added for 24 h. Cell viability was determined by using an MTT assay kit (Millipore) according to the manufacturer's instructions.

For analysis of spheroid formation of HCT116 cells, the shRNA-resistant expression vector of FLAG-PGRMC1 or an empty vector was transfected as described above. After 24 h, cells were seeded at 1 × 10^4^ cells per well onto a 96-well spheroid culture plate (NanoCulture plate with a microsquare pattern, SCIVAX Corp.) and incubated for three days. The size of individual spheroid was determined by measuring their optical areas using Image-J and by calculating the apparent radius (*r*) to estimate their apparent volume (*v*) according to the following formula: *v*=4/3 × *πr*^3^.

### Measurements of intracellular haem concentrations

To measure protohaem (haem *b*) concentrations, LC-UV and LC-MS analyses were performed for quantification and molecular identification, respectively. Briefly, HCT116 cells (1 × 10^7^ cells) were treated with vehicle or 250 μmol l^−1^ SA for 48 h. After centrifugation, haem *b* was extracted from cell pellets twice by adding acetone containing 30% formate, followed by a 5 min sonication and centrifugation. The supernatant was collected, and the solvent was evaporated. The dried residues were re-dissolved in acetonitrile containing 0.2% formate and subjected to a LCMS-8030 system equipped with photodiode array (PDA) detector (SPD-20A) (Shimadzu Corporation, Kyoto, Japan). Haem *b* was detected by monitoring the absorption at 400 nm. Its identity was confirmed by simultaneous mass spectrometric analysis at *m/z* 616.

### Analyses of doxorubicin metabolism

HCT116 cells (5 × 10^6^ cells/10 cm dish) were cultured for 2 days, after which the cells were cultured in the presence of 0.3 μmol l^−1^ doxorubicin overnight. The cells were lysed with methanol containing internal standard compounds, and then water-soluble fraction was separated by liquid-liquid extraction (chloroform: methanol: water=1: 2: 1). The amounts of doxorubicin and its metabolites were quantified using LC-MS/MS. Briefly, a triple-quadrupole mass spectrometer equipped with an electrospray ionization (ESI) ion source (LCMS-8030; Shimadzu Corporation) was used in the positive-ESI and multiple reaction monitoring modes. The samples were resolved on an ACQUITY UPLC BEH C18 column (100 × 2.1 mm i.d., 1.7 μm particle) using water and acetonitrile as mobile phases A and B, respectively, at a flow rate of 0.15 ml min^−1^ and a column temperature of 40 °C. Ion transitions from *m/z* 544 to *m/z* 130 and from *m/z* 546 to *m/z* 399 for doxorubicin and doxorubicinol, respectively, were monitored for their quantification.

### Xenograft implantation of HCT116 cells

All the protocols for animal experiments in this study were approved by the Experimental Animal Committee of Keio University School of Medicine (the approved number; 08037-(7)). A model of liver metastases of human colon cancer was prepared according to our previous methods with minor modifications^7^,^30^. Briefly, HCT116 cells transfected with the cDNA of Venus (1 × 10^6^ cells/mice), a highly sensitive fluorescent protein on tissue slice sections,^66^ were transplanted into the spleens of 10-week-old male NOG mice. Ten days after transplantation, the animals were anesthetized with sevoflurane (Maruishi Pharmaceutical), and their livers were removed, embedded in super-cryoembedding medium and frozen quickly in liquid nitrogen. The frozen tissues were sliced with a cryostat (Leica CM1900) at a thickness of 5 μm, and haematoxylin-eosin staining was performed. Fluorescent microscopy (Keyence: BIOREVO, BZ-9000) was used to observe Venus fluorescence. Percentages of cross-sectional areas showing metastatic tumours were calculated by Image-J as previously described^7^.

## Additional information

**Accession code:** Structural information on PGRMC1 is available from the Protein Data Bank under accession code 4X8Y.

**How to cite this article:** Kabe, Y. *et al*. Haem-dependent dimerization of PGRMC1/sigma-2 receptor facilitates cancer proliferation and chemoresistance. *Nat. Commun.* 7:11030 doi: 10.1038/ncomms11030 (2016).

## Supplementary Material

Supplementary Supplementary Figures 1-21, Supplementary Table 1, Supplementary Methods and Supplementary References.

## Figures and Tables

**Table 1:** Data collection and refinement statistics.

	**Native**	**Phasing**
*Data collection*		
Space group	*I*4_1_22	*I*4_1_22
Cell dimensions		
*a, b, c* (Å)	167.23, 167.23, 63.46	168.11, 168.11, 63.65
*α, β, γ* (°)	90, 90, 90	90, 90, 90
Wavelength (Å)	1.000	1.738
Resolution (Å)	20.0–1.95 (2.00–1.95)	20.0–2.50 (2.56–2.50)
*R*_meas_	0.067 (1.168)	0.010 (0.850)
I/σI	22.83 (2.39)	22.43 (4.54)
Completeness (%)	97.8 (99.0)	99.1 (97.6)
Multiplicity	11.2 (13.8)	14.9 (15.2)
CC1/2	100 (81.8)	99.9 (93.5)
		
*Refinement*		
Resolution (Å)	19.72–1.95	
Number of reflections	32,298 (2,384)	
*R*_work_/*R*_free_	0.1834/0.2123	
Number of atoms		
Protein	1,776	
Ligand/ion	86	
Water	109	
B-factors		
Protein	54.6	
Ligand/ion	42.9	
Water	46.6	
R.M.S deviations		
Bond lengths (Å)	0.008	
Bond angles (°)	1.164	

*Highest resolution shell is shown in parenthesis.

**Table 2:** PGRMC1 proteins exhibit haem-dependent dimerization in solution.

	**Apo form**		**Haem-bound form**	
		Mass (Da)		Mass (Da)
**a** *PGRMC1 wt (a.a.44–195)*				
ESI-MS	—	17,844.14	—	36,920.19
Theoretical		17,843.65		36,918.06
	Hydrodynamic radius 10^−9^ (m)	MW (kDa)	Hydrodynamic radius 10^−9^ (m)	MW (kDa)
DOSY	2.04–2.15	20	2.94–3.02	42
	*S*_20,*w*_ (S)	MW (kDa)	*S*_20,*w*_ (S)	MW (kDa)
SV-AUC	1.9	17.6	3.1	35.5
				
**b** *PGRMC1 C129S (a.a.44–195)*				
ESI-MS	—	17,827.91	—	36,887.07
Theoretical		17,827.59		36,885.6
	*S*_20,*w*_ (S)	MW (kDa)	*S*_20,*w*_ (S)	MW (kDa)
SV-AUC	2.0	18.1	3.1	35.8

Differences in molecular weights of the wild-type (wt; **a**) and the C129S mutant (**b**) PGRMC1 proteins in the absence (apo form) or the presence of haem (haem-bound form). The protein sizes of the wt and C129S PGRMC1 cytosolic domains (a.a.44–195) in the presence or absence of haem were estimated by ESI-MS, DOSY and SV-AUC.

## References

[b1] Kowdley K. V. Iron, hemochromatosis, and hepatocellular carcinoma. Gastroenterology 127, S79–S86 (2004). 15508107 10.1016/j.gastro.2004.09.019

[b2] Jakszyn P. . Meat and heme iron intake and esophageal adenocarcinoma in the European prospective investigation into cancer and nutrition study. Int. J. Cancer. 133, 2744–2750 (2013). 10.1002/ijc.28291 23728954

[b3] Bastide N. M., Pierre F. H. & Corpet D. E. Heme iron from meat and risk of colorectal cancer: a meta-analysis and a review of the mechanisms involved. Cancer Prev. Res. (Phila.) 4, 177–184 (2011). 21209396 10.1158/1940-6207.CAPR-10-0113

[b4] Shen J. . Iron metabolism regulates p53 signaling through direct heme-p53 interaction and modulation of p53 localization, stability and function. Cell Rep. 7, 180–193 (2014). 24685134 10.1016/j.celrep.2014.02.042 PMC4219651

[b5] Hooda J. . Enhanced heme function and mitochondrial respiration promote the progression of lung cancer cells. PLoS ONE 8, e63402 (2013). 23704904 10.1371/journal.pone.0063402 PMC3660535

[b6] Wegiel B. . Carbon monoxide expedites metabolic exhaustion to inhibit tumor growth. Cancer Res. 73, 7009–7021 (2013). 24121491 10.1158/0008-5472.CAN-13-1075 PMC3851591

[b7] Yamamoto T. . Reduced methylation of PFKFB3 in cancer cells shunts glucose towards the pentose phosphate pathway. Nat. Commun. 5, 3480 (2014). 24633012 10.1038/ncomms4480 PMC3959213

[b8] Sakamoto S., Kabe Y., Hatakeyama M., Yamaguchi Y. & Handa H. Development and application of high-performance affinity beads: toward chemical biology and drug discovery. Chem. Rec. 9, 66–85 (2009). 19243077 10.1002/tcr.20170

[b9] Mifsud W. & Bateman A. Membrane-bound progesterone receptors contain a cytochrome *b*_5_-like ligand-binding domain. Genome. Biol. 3, RESEARCH0068 (2002). 12537557 10.1186/gb-2002-3-12-research0068 PMC151170

[b10] Neubauer H. . Possible role of PGRMC1 in breast cancer development. Climacteric 16, 509–513 (2013). 23758160 10.3109/13697137.2013.800038

[b11] Craven R. J. PGRMC1: a new biomarker for the estrogen receptor in breast cancer. Breast Cancer Res. 10, 113 (2008). 19090968 10.1186/bcr2191 PMC2656908

[b12] Peluso J. J., Liu X., Saunders M. M., Claffey K. P. & Phoenix K. Regulation of ovarian cancer cell viability and sensitivity to cisplatin by progesterone receptor membrane component-1. J. Clin. Endocrinol. Metab. 93, 1592–1599 (2008). 18319313 10.1210/jc.2007-2771

[b13] Friel A. M. . Progesterone receptor membrane component 1 deficiency attenuates growth while promoting chemosensitivity of human endometrial xenograft tumors. Cancer Lett. 356, 434–442 (2015). 25304370 10.1016/j.canlet.2014.09.036 PMC4259802

[b14] Nie A. Y. . Predictive toxicogenomics approaches reveal underlying molecular mechanisms of nongenotoxic carcinogenicity. Mol. Carcinog. 45, 914–933 (2006). 16921489 10.1002/mc.20205

[b15] Mir S. U., Ahmed I. S., Arnold S. & Craven R. J. Elevated progesterone receptor membrane component 1/sigma-2 receptor levels in lung tumors and plasma from lung cancer patients. Int. J. Cancer 131, E1–E9 (2012). 21918976 10.1002/ijc.26432

[b16] Hornick J. R., Spitzer D., Goedegebuure P., Mach R. H. & Hawkins W. G. Therapeutic targeting of pancreatic cancer utilizing sigma-2 ligands. Surgery 152, S152–S156 (2012). 22763259 10.1016/j.surg.2012.05.014 PMC3982787

[b17] Crudden G., Loesel R. & Craven R. J. Overexpression of the cytochrome p450 activator hpr6 (heme-1 domain protein/human progesterone receptor) in tumors. Tumour Biol. 26, 142–146 (2005). 15970648 10.1159/000086485

[b18] Ahmed I. S., Rohe H. J., Twist K. E. & Craven R. J. Pgrmc1 (progesterone receptor membrane component 1) associates with epidermal growth factor receptor and regulates erlotinib sensitivity. J. Biol. Chem. 285, 24775–24782 (2010). 20538600 10.1074/jbc.M110.134585 PMC2915713

[b19] Hughes A. L. . Dap1/PGRMC1 binds and regulates cytochrome P450 enzymes. Cell Metab. 5, 143–149 (2007). 17276356 10.1016/j.cmet.2006.12.009

[b20] Ahmed I. S., Rohe H. J., Twist K. E., Mattingly M. N. & Craven R. J. Progesterone receptor membrane component 1 (Pgrmc1): a heme-1 domain protein that promotes tumorigenesis and is inhibited by a small molecule. J. Pharmacol. Exp. Ther. 333, 564–573 (2010). 20164297 10.1124/jpet.109.164210

[b21] Ahmed I. S., Chamberlain C. & Craven R. J. S2R(Pgrmc1): the cytochrome-related sigma-2 receptor that regulates lipid and drug metabolism and hormone signaling. Expert Opin. Drug. Metab. Toxicol. 8, 361–370 (2012). 22292588 10.1517/17425255.2012.658367

[b22] Kaluka D., Batabyal D., Chiang B. Y., Poulos T. L. & Yeh S. R. Spectroscopic and mutagenesis studies of human PGRMC1. Biochemistry 54, 1638–1647 (2015). 25675345 10.1021/bi501177e PMC4533898

[b23] Min L. . Molecular identification of adrenal inner zone antigen as a heme-binding protein. FEBS J. 272, 5832–5843 (2005). 16279947 10.1111/j.1742-4658.2005.04977.x

[b24] Krissinel E. & Henrick K. Inference of macromolecular assemblies from crystalline state. J. Mol. Biol. 372, 774–797 (2007). 17681537 10.1016/j.jmb.2007.05.022

[b25] Shen Y., Delaglio F., Cornilescu G. & Bax A. TALOS+: a hybrid method for predicting protein backbone torsion angles from NMR chemical shifts. J. Biomol. NMR. 44, 213–223 (2009). 19548092 10.1007/s10858-009-9333-z PMC2726990

[b26] Ohto U. . Structural basis of CpG and inhibitory DNA recognition by Toll-like receptor 9. Nature 520, 702–705 (2015). 25686612 10.1038/nature14138

[b27] Nogi T. . Structural basis for semaphorin signalling through the plexin receptor. Nature 467, 1123–1127 (2010). 20881961 10.1038/nature09473

[b28] Ishikawa H. . Involvement of heme regulatory motif in heme-mediated ubiquitination and degradation of IRP2. Mol. Cell. 19, 171–181 (2005). 16039587 10.1016/j.molcel.2005.05.027

[b29] Liu Y. . Replacement of the proximal histidine iron ligand by a cysteine or tyrosine converts heme oxygenase to an oxidase. Biochemistry 38, 3733–3743 (1999). 10090762 10.1021/bi982707s

[b30] Kubo A. . Semi-quantitative analyses of metabolic systems of human colon cancer metastatic xenografts in livers of superimmunodeficient NOG mice. Anal. Bioanal. Chem. 400, 1895–1904 (2011). 21479793 10.1007/s00216-011-4895-5 PMC3098365

[b31] Bao Y. . Energy management by enhanced glycolysis in G1-phase in human colon cancer cells in vitro and in vivo. Mol. Cancer Res. 11, 973–985 (2013). 23741060 10.1158/1541-7786.MCR-12-0669-T

[b32] Quintieri L. . In vivo antitumor activity and host toxicity of methoxymorpholinyl doxorubicin: role of cytochrome P450 3A. Cancer Res. 60, 3232–3238 (2000). 10866316

[b33] McFadyen M. C. . Cytochrome P450 CYP1B1 protein expression: a novel mechanism of anticancer drug resistance. Biochem. Pharmacol. 62, 207–212 (2001). 10.1016/s0006-2952(01)00643-8 11389879

[b34] Szczesna-Skorupa E. & Kemper B. Progesterone receptor membrane component 1 inhibits the activity of drug-metabolizing cytochromes P450 and binds to cytochrome P450 reductase. Mol. Pharmacol. 79, 340–350 (2011). 21081644 10.1124/mol.110.068478 PMC3061357

[b35] Higashimoto Y. . Involvement of NADPH in the interaction between heme oxygenase-1 and cytochrome P450 reductase. J. Biol. Chem. 280, 729–737 (2005). 15516695 10.1074/jbc.M406203200

[b36] Wakasugi K., Nakano T. & Morishima I. Oxidized human neuroglobin acts as a heterotrimeric Galpha protein guanine nucleotide dissociation inhibitor. J. Biol. Chem. 278, 36505–36512 (2003). 12860983 10.1074/jbc.M305519200

[b37] Lucas A. T. . Ligand binding reveals a role for heme in translationally-controlled tumor protein dimerization. PLoS ONE 9, e112823 (2014). 25396429 10.1371/journal.pone.0112823 PMC4232476

[b38] Watanabe M. . Structural basis for multimeric heme complexation through a specific protein-heme interaction: the case of the third neat domain of IsdH from Staphylococcus aureus. J. Biol. Chem. 283, 28649–28659 (2008). 18667422 10.1074/jbc.M803383200 PMC2661414

[b39] Cahill M. A. Progesterone receptor membrane component 1: an integrative review. J. Steroid Biochem. Mol. Biol. 105, 16–36 (2007). 17583495 10.1016/j.jsbmb.2007.02.002

[b40] Peluso J. J., Griffin D., Liu X. & Horne M. Progesterone receptor membrane component-1 (PGRMC1) and PGRMC-2 interact to suppress entry into the cell cycle in spontaneously immortalized rat granulosa cells. Biol. Reprod. 91, 104 (2014). 25253729 10.1095/biolreprod.114.122986 PMC4434922

[b41] Craven R. J., Mallory J. C. & Hand R. A. Regulation of iron homeostasis mediated by the heme-binding protein Dap1 (damage resistance protein 1) via the P450 protein Erg11/Cyp51. J. Biol. Chem. 282, 36543–36551 (2007). 17954932 10.1074/jbc.M706770200

[b42] Oda S., Nakajima M., Toyoda Y., Fukami T. & Yokoi T. Progesterone receptor membrane component 1 modulates human cytochrome P450 activities in an isoform-dependent manner. Drug Metab. Dispos. 39, 2057–2065 (2011). 21825115 10.1124/dmd.111.040907

[b43] Lin S. T. . PGRMC1 contributes to doxorubicin-induced chemoresistance in MES-SA uterine sarcoma. Cell. Mol. Life Sci. 72, 2395–2409 (2015). 25596698 10.1007/s00018-014-1831-9 PMC11397629

[b44] Wendler A., Keller D., Albrecht C., Peluso J. J. & Wehling M. Involvement of let-7/miR-98 microRNAs in the regulation of progesterone receptor membrane component 1 expression in ovarian cancer cells. Oncol. Rep. 25, 273–279 (2011). 21109987

[b45] Liu N., Zhou C., Zhao J. & Chen Y. Reversal of paclitaxel resistance in epithelial ovarian carcinoma cells by a MUC1 aptamer-let-7i chimera. Cancer Invest. 30, 577–582 (2012). 22812695 10.3109/07357907.2012.707265

[b46] Kajimura M., Fukuda R., Bateman R. M., Yamamoto T. & Suematsu M. Interactions of multiple gas-transducing systems: hallmarks and uncertainties of CO, NO, and H_2_S gas biology. Antioxid. Redox. Signal. 13, 157–192 (2010). 19939208 10.1089/ars.2009.2657 PMC2925289

[b47] Kyokane T. . Carbon monoxide from heme catabolism protects against hepatobiliary dysfunction in endotoxin-treated rat liver. Gastroenterology 120, 1227–1240 (2001). 11266386 10.1053/gast.2001.23249

[b48] Suematsu M. & Ishimura Y. The heme oxygenase-carbon monoxide system: a regulator of hepatobiliary function. Hepatology 31, 3–6 (2000). 10613719 10.1002/hep.510310102

[b49] Chen C. & Paw B. H. Cellular and mitochondrial iron homeostasis in vertebrates. Biochim. Biophys. Acta. 1823, 1459–1467 (2012). 22285816 10.1016/j.bbamcr.2012.01.003 PMC3350831

[b50] Frezza C. . Haem oxygenase is synthetically lethal with the tumour suppressor fumarate hydratase. Nature 477, 225–228 (2011). 21849978 10.1038/nature10363

[b51] Lee W. Y. . The induction of heme oxygenase-1 suppresses heat shock protein 90 and the proliferation of human breast cancer cells through its byproduct carbon monoxide. Toxicol. Appl. Pharmacol. 274, 55–62 (2014). 24211270 10.1016/j.taap.2013.10.027

[b52] Xu J. . Identification of the PGRMC1 protein complex as the putative sigma-2 receptor binding site. Nat. Commun. 2, 380 (2011). 21730960 10.1038/ncomms1386 PMC3624020

[b53] Izzo N. J. . Alzheimer's therapeutics targeting amyloid beta 1-42 oligomers II: Sigma-2/PGRMC1 receptors mediate Abeta 42 oligomer binding and synaptotoxicity. PLoS ONE 9, e111899 (2014). 25390692 10.1371/journal.pone.0111899 PMC4229119

[b54] Qin Y. . Progesterone attenuates Abeta25-35-induced neuronal toxicity via JNK inactivation and progesterone receptor membrane component 1-dependent inhibition of mitochondrial apoptotic pathway. J. Steroid Biochem. Mol. Biol. 154, 302–311 (2015). 25576906 10.1016/j.jsbmb.2015.01.002

[b55] Sahlholm K., Liao F., Holtzman D. M., Xu J. & Mach R. H. Sigma-2 receptor binding is decreased in female, but not male, APP/PS1 mice. Biochem. Biophys. Res. Commun. 460, 439–445 (2015). 25796326 10.1016/j.bbrc.2015.03.052 PMC6818091

[b56] Kabsch W. XDS. Acta. Crystallogr. D Biol. Crystallogr. 66, 125–132 (2010). 20124692 10.1107/S0907444909047337 PMC2815665

[b57] Terwilliger T. C. . Decision-making in structure solution using Bayesian estimates of map quality: the PHENIX AutoSol wizard. Acta. Crystallogr. D Biol. Crystallogr. 65, 582–601 (2009). 19465773 10.1107/S0907444909012098 PMC2685735

[b58] Emsley P. & Cowtan K. Coot: model-building tools for molecular graphics. Acta. Crystallogr. D Biol. Crystallogr. 60, 2126–2132 (2004). 15572765 10.1107/S0907444904019158

[b59] Adams P. D. . PHENIX: a comprehensive Python-based system for macromolecular structure solution. Acta. Crystallogr. D Biol. Crystallogr. 66, 213–221 (2010). 20124702 10.1107/S0907444909052925 PMC2815670

[b60] Schuck P. Size-distribution analysis of macromolecules by sedimentation velocity ultracentrifugation and lamm equation modeling. Biophys. J. 78, 1606–1619 (2000). 10692345 10.1016/S0006-3495(00)76713-0 PMC1300758

[b61] Brookes E., Demeler B., Rosano C. & Rocco M. The implementation of SOMO (SOlution MOdeller) in the UltraScan analytical ultracentrifugation data analysis suite: enhanced capabilities allow the reliable hydrodynamic modeling of virtually any kind of biomacromolecule. Eur. Biophys. J. 39, 423–435 (2010). 19234696 10.1007/s00249-009-0418-0 PMC2872189

[b62] Wu D. H., Chen A. D. & Johnson C. S. An improved diffusion-ordered spectroscopy experiment incorporating bipolar-gradient pulses. J. Magn. Reson. A. 115, 260–264 (1995).

[b63] Esturau N. . The use of sample rotation for minimizing convection effects in self-diffusion NMR measurements. J. Magn. Reson. 153, 48–55 (2001). 11700080 10.1006/jmre.2001.2411

[b64] Suematsu M. . Carbon monoxide: an endogenous modulator of sinusoidal tone in the perfused rat liver. J. Clin. Invest. 96, 2431–2437 (1995). 7593631 10.1172/JCI118300 PMC185895

[b65] Goda N. . Distribution of heme oxygenase isoforms in rat liver. Topographic basis for carbon monoxide-mediated microvascular relaxation. J. Clin. Invest. 101, 604–612 (1998). 9449694 10.1172/JCI1324 PMC508604

[b66] Nagai T. . A variant of yellow fluorescent protein with fast and efficient maturation for cell-biological applications. Nat. Biotechnol. 20, 87–90 (2002). 11753368 10.1038/nbt0102-87

