# Supplementary material for: Haem-dependent dimerization of PGRMC1/Sigma-2 receptor facilitates cancer proliferation and chemoresistance
Source: Nat Commun. 2016 Mar 18;7:11030. doi: 10.1038/ncomms11030 (PMC4802085; doi:10.1038/ncomms11030)
Supplement: Supplementary — Figures 1-21, Supplementary Table 1, Supplementary Methods and Supplementary References. [file ncomms11030-s1.pdf]

Supplementary Information

SUPPLEMENTARY FIGURES

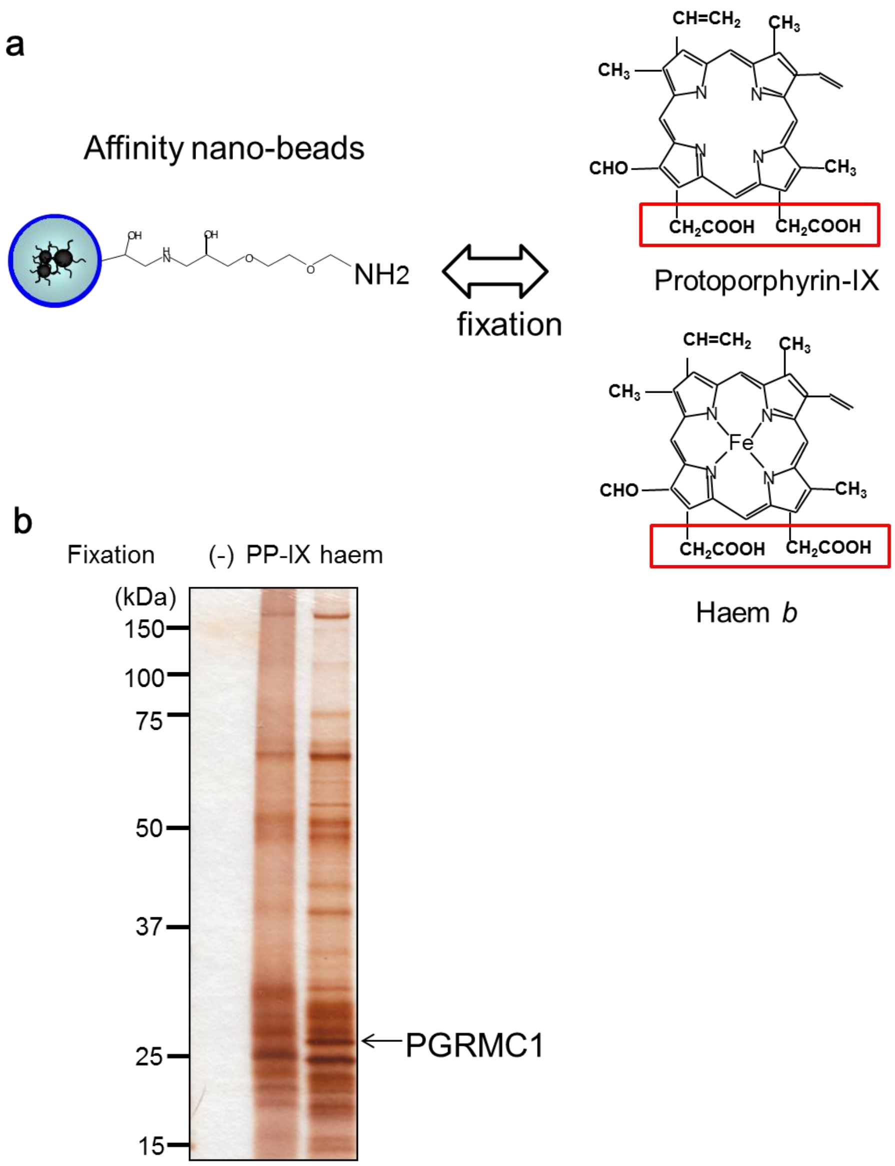

Supplementary Figure 1 Affinity purification of PGRMC1 with haem-fixed nano-beads

(**a**) A scheme for fixation of protoporphyrin-IX (PP-IX) and haem with amino-modified affinity beads. (**b**) Control, PP-IX- or haem-fixed beads were incubated with mouse liver lysates. PGRMC1 was identified by peptide sequence with ESI-MS.

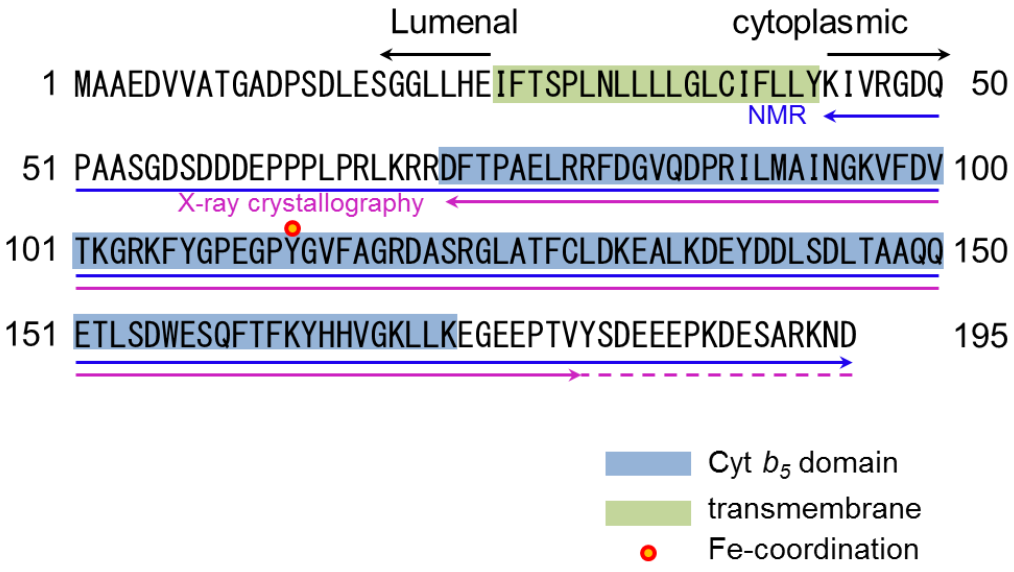

Supplementary Figure 2 Primary amino acid sequence of human PGRMC1

Violet and cyan arrows show regions of the recombinant protein on X-ray crystallography and NMR analysis, respectively.

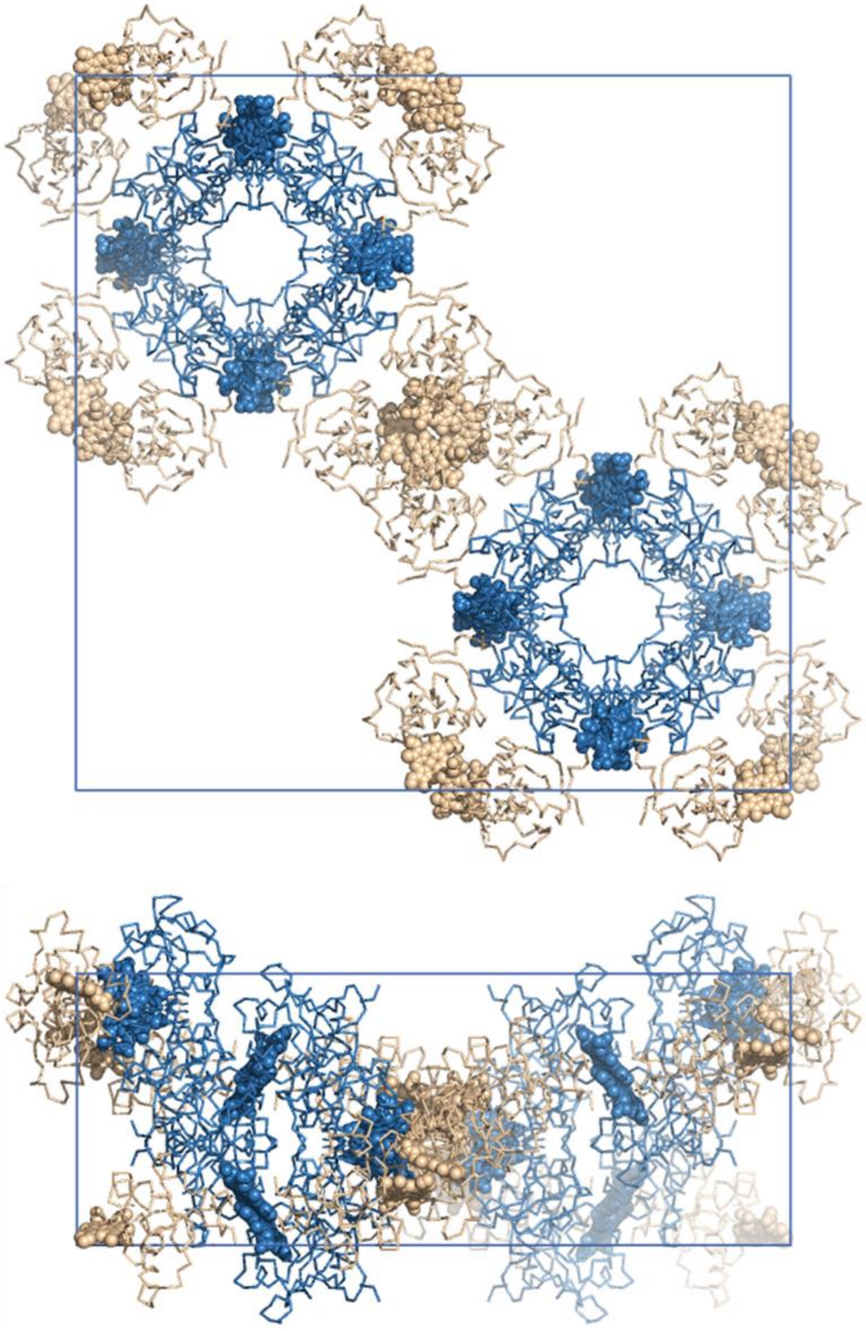

Supplementary Figure 3 Crystal packing of PGRMC1

(**a**) Crystal packing in the a-b plane (left panel) and the a-c plane (right panel). Chains A and B are coloured blue and gold, respectively. The unit cell is drawn blue. (**b**) 2Fo-Fc electron density map around the haem is shown in gray mesh, contoured at 1.5 σ. For clarity, the map is carved at 3 Å from the haem and Tyr113.

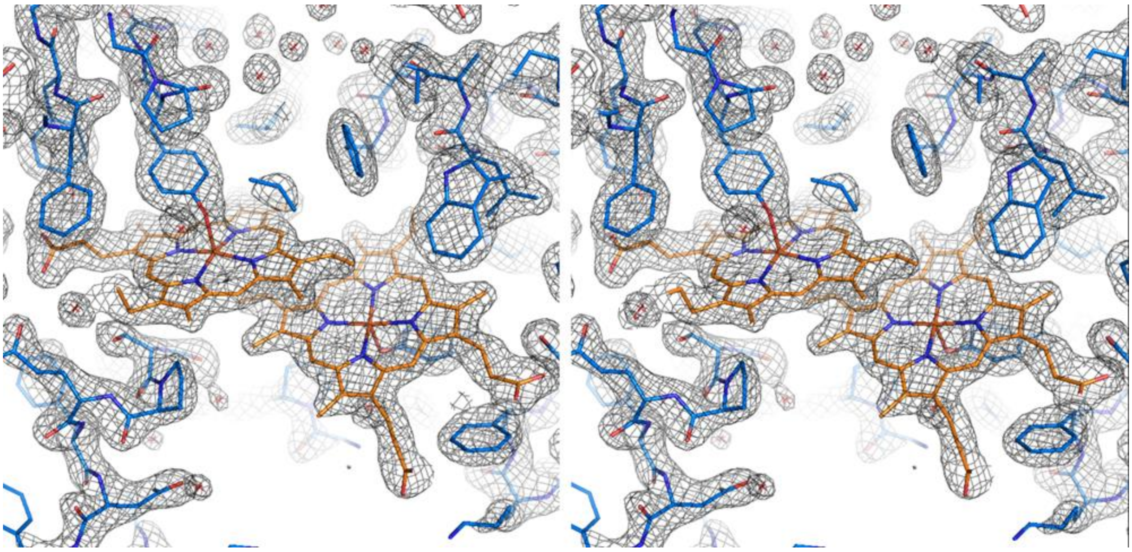

Supplementary Figure 4

Stereo view of density for the haem-haem stacked region of PGMRC1

The 2mFo-DFc density map was contoured at 2 sigma.

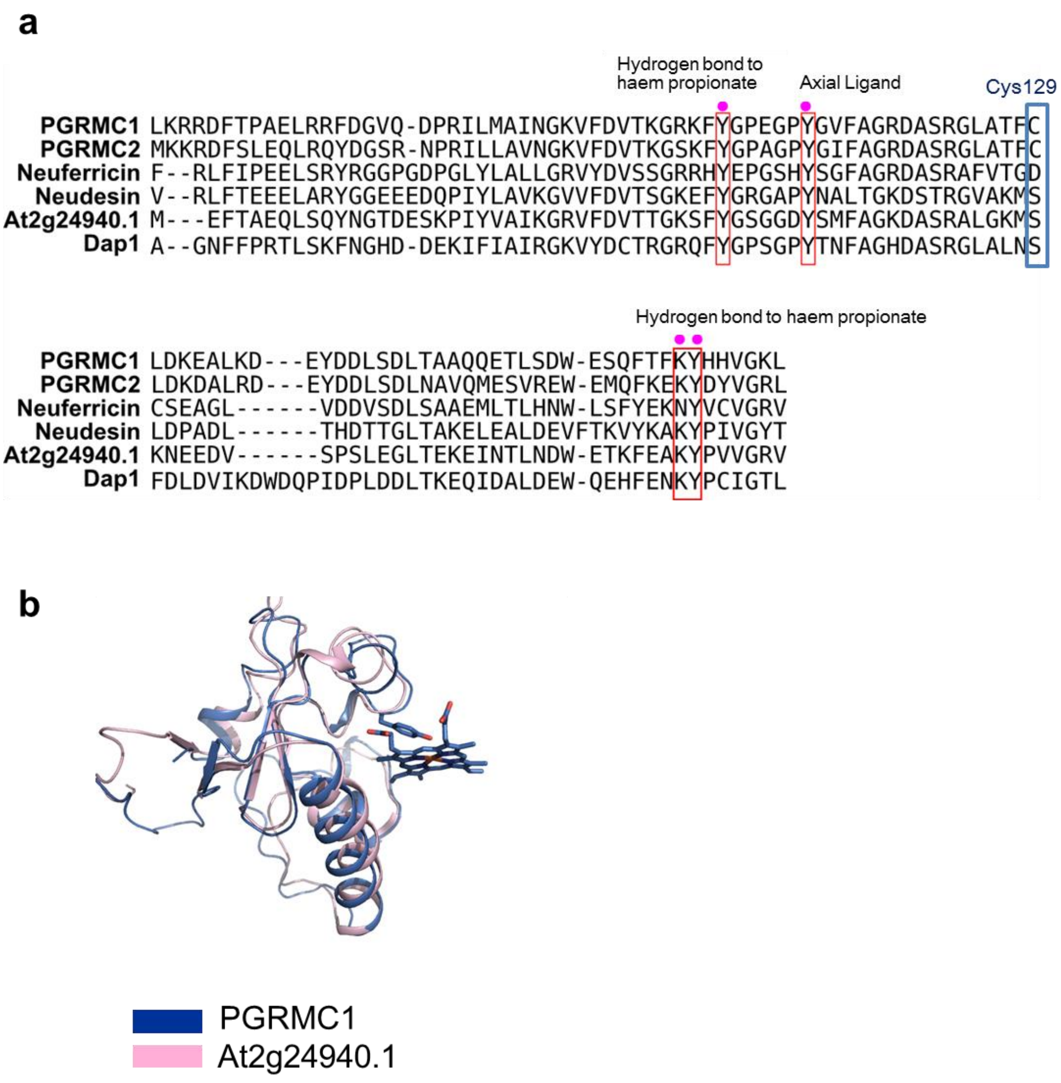

Supplementary Figure 5

Sequence alignment and structure comparison with PGRMC1 homologues

(**a**) Sequence alignment of cytochrome b_5-like domains of MAPR family members and canonical cytochrome b_5. MAPR members share unique haem-binding residues that are not present in canonical cytochrome b_5. (**b**) Structure comparison between PGRMC1 (blue) and Arabidopsis thaliana homologue At2g24940.1 (pink). The latter structure (PDBID: 1T0G) was determined with NMR in its apo state.

a

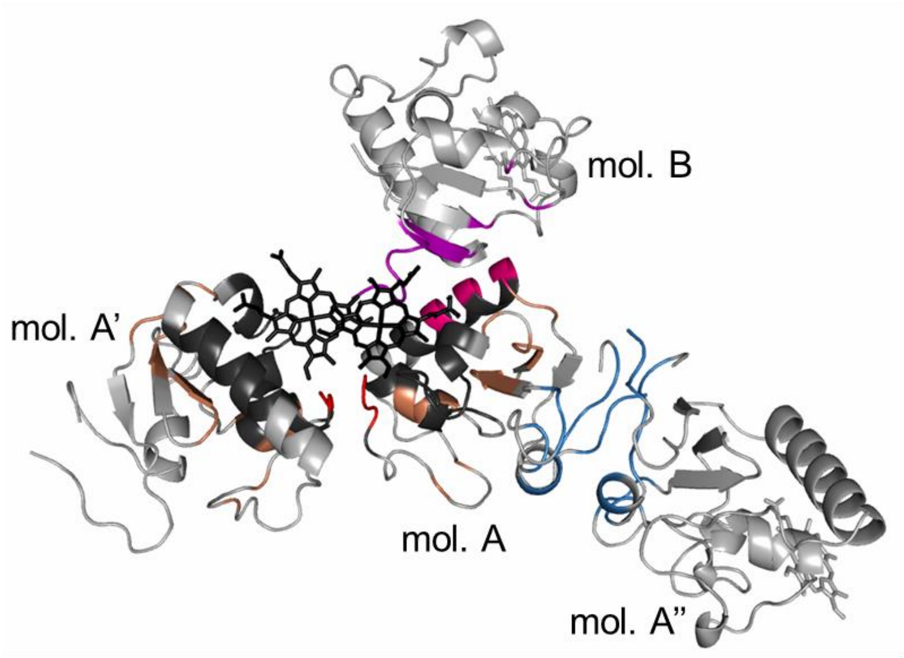
mol. B
mol. A'
mol. A
mol. A"

b

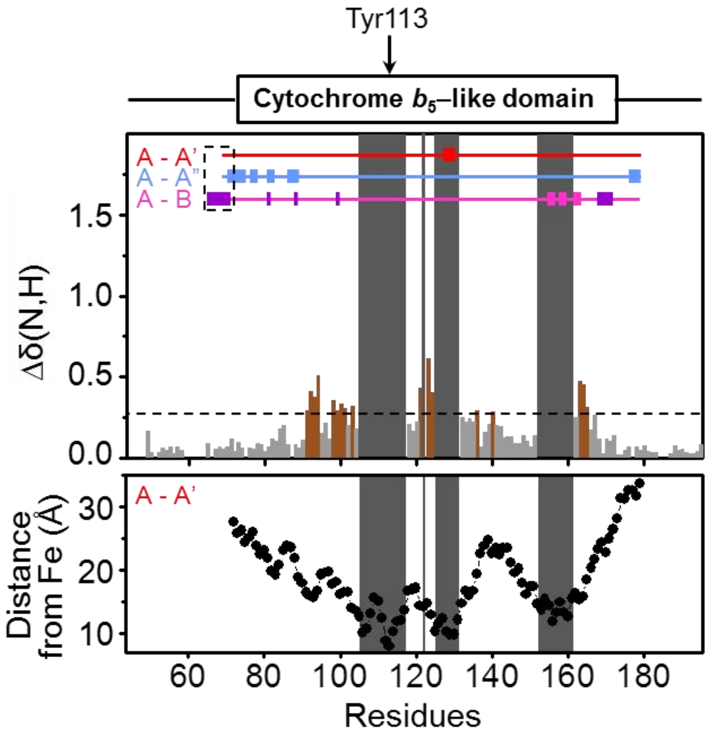
Tyr113
Cytochrome b5–like domain
A - A'
A - A"
A - B
Δδ(N,H)
1.5
1.0
0.5
0.0
A - A'
Distance from Fe (Å)
30
20
10
60
80
100
120
140
160
180
Residues

Supplementary Figure 6 Structural analysis of PGRMC1 by NMR

(**a**) PGRMC1 molecule (mol. A) directly interacts with three molecules (A', A" and B) in the crystal lattice. Red, violet, pink, and cyan residues show direct contacts with neighbouring molecules. Black residues show the region of the ^{1}H-^{15}N chemical shift that disappeared due to the paramagnetic relaxation effect of haem iron in NMR analysis. Regions highlighted in dark yellow show amino acid residues showing the notable differences in ^{1}H-^{15}N chemical shift ($\Delta\delta$ (N, H)) that are greater than the standard deviation (SD) of the chemical shifts in response to haem binding that are shown in Supplementary Fig. 6b.

(**b**) Differences in the ^{1}H-^{15}N chemical shift ($\Delta\delta$ (N, H)) reflecting the changes in environments for individual residues of PGRMC1 (a.a.44-195) in response to haem binding. The broken line shows the standard deviation (SD) of $\Delta\delta$ (N, H). Bars highlighted in dark yellow show amino acid residues indicating greater differences in the chemical shit than the SD value. The dark gray regions show residues corresponding to signals that disappeared due to the paramagnetic relaxation effect caused by haem iron. Filled boxes on the three crossbars represent direct interaction residues in the crystal lattice (corresponding to the colours used in Supplementary Fig. 3a). The dashed box highlights the region of the tag sequence from the vector. Lower panel shows distance from Fe atoms of haem to individual amide nitrogens in A-A' dimer structure.

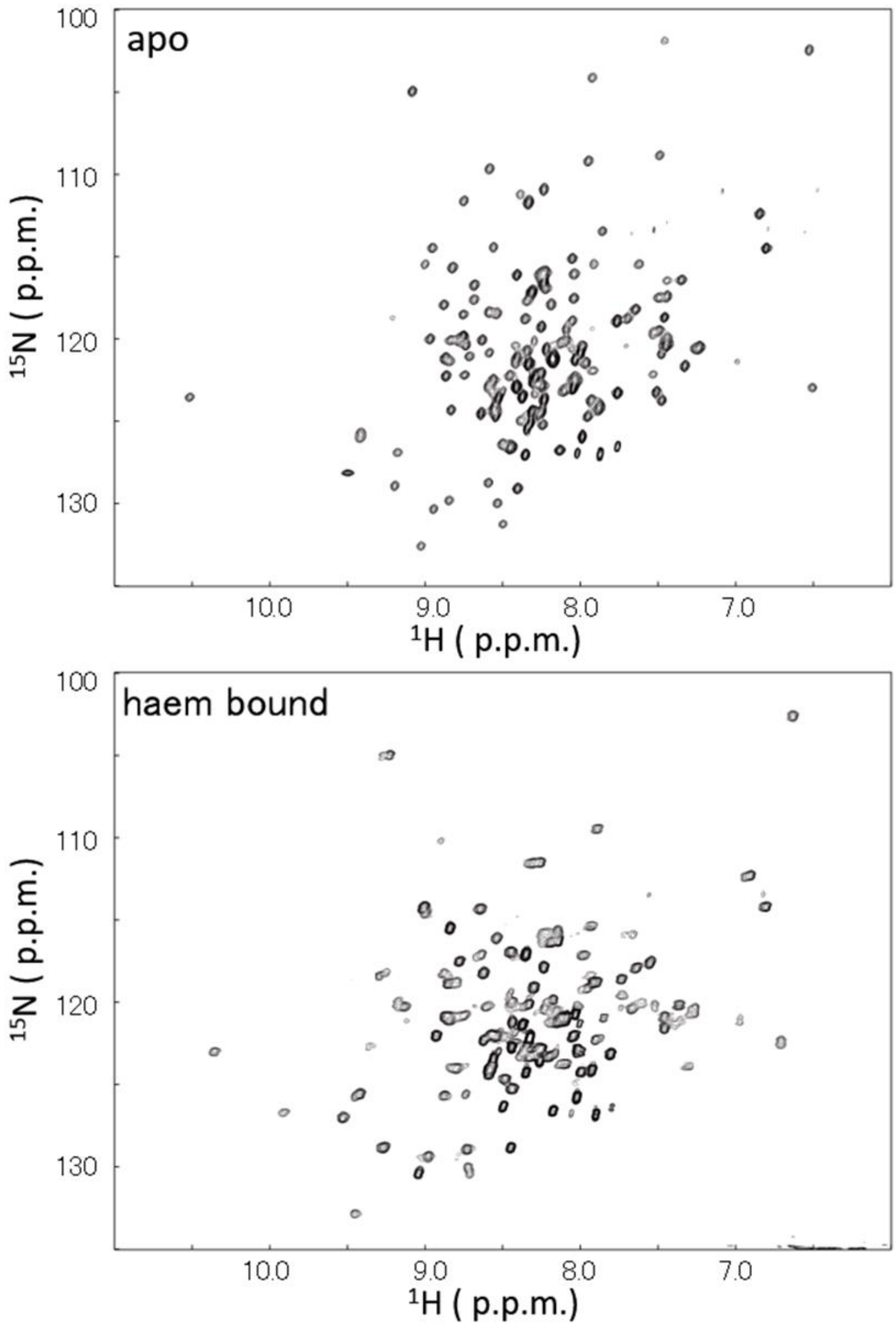

Supplementary Figure 7 NMR spectrum of PGRMC1

Raw data of the [^{15}N, ^{1}H]-TROSY spectra of the monomer (apo form: upper panel) and dimer (heam-bound form: lower panel) of PGRMC1 (a.a.44-195)

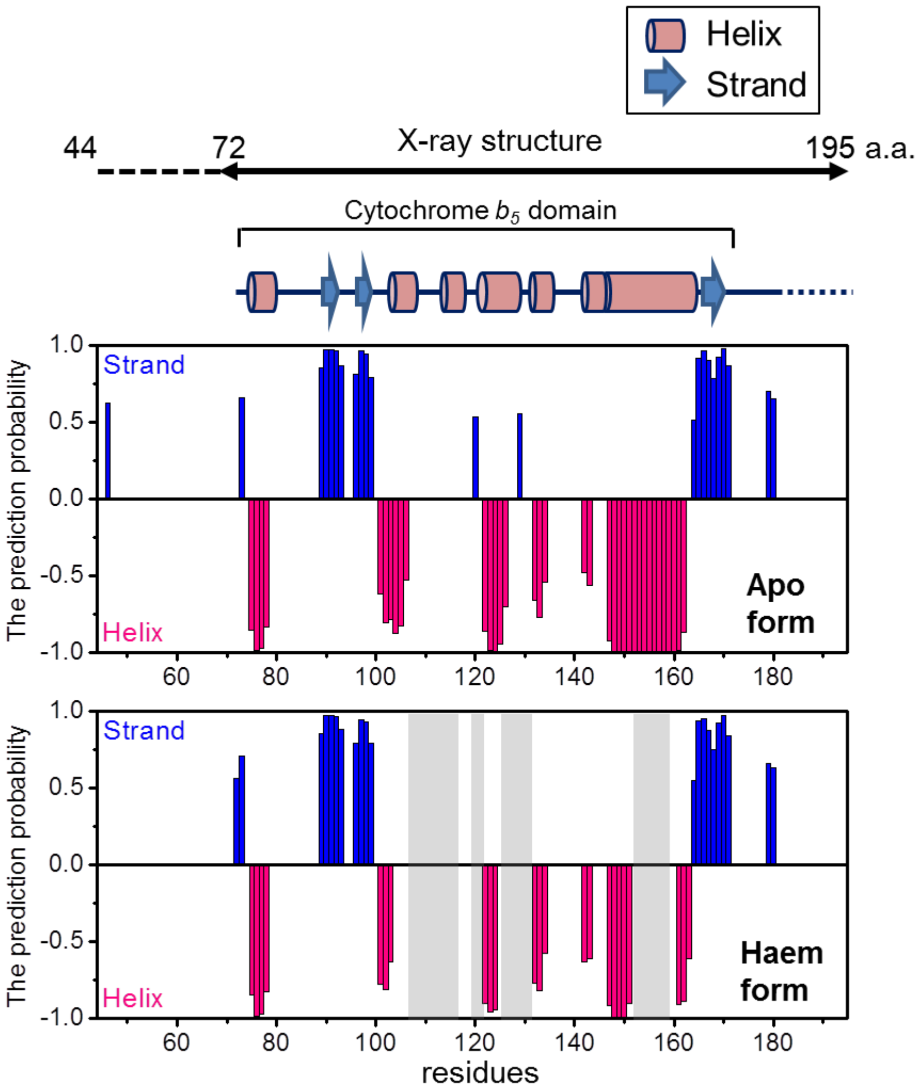

Supplementary Figure 8 Prediction of secondary structure of PGRMC1

The secondary structure of the apo- or haem-form of PGRMC1 was predicted by calculating the NMR data with TALOS+ program, and compared with X-ray crystallographic data. The length of the bars corresponds to the prediction probability of a residue to be helix or β-strand. Gray regions show the residues undetected because of effects of paramagnetic iron of the haem-form of PGRMC1.

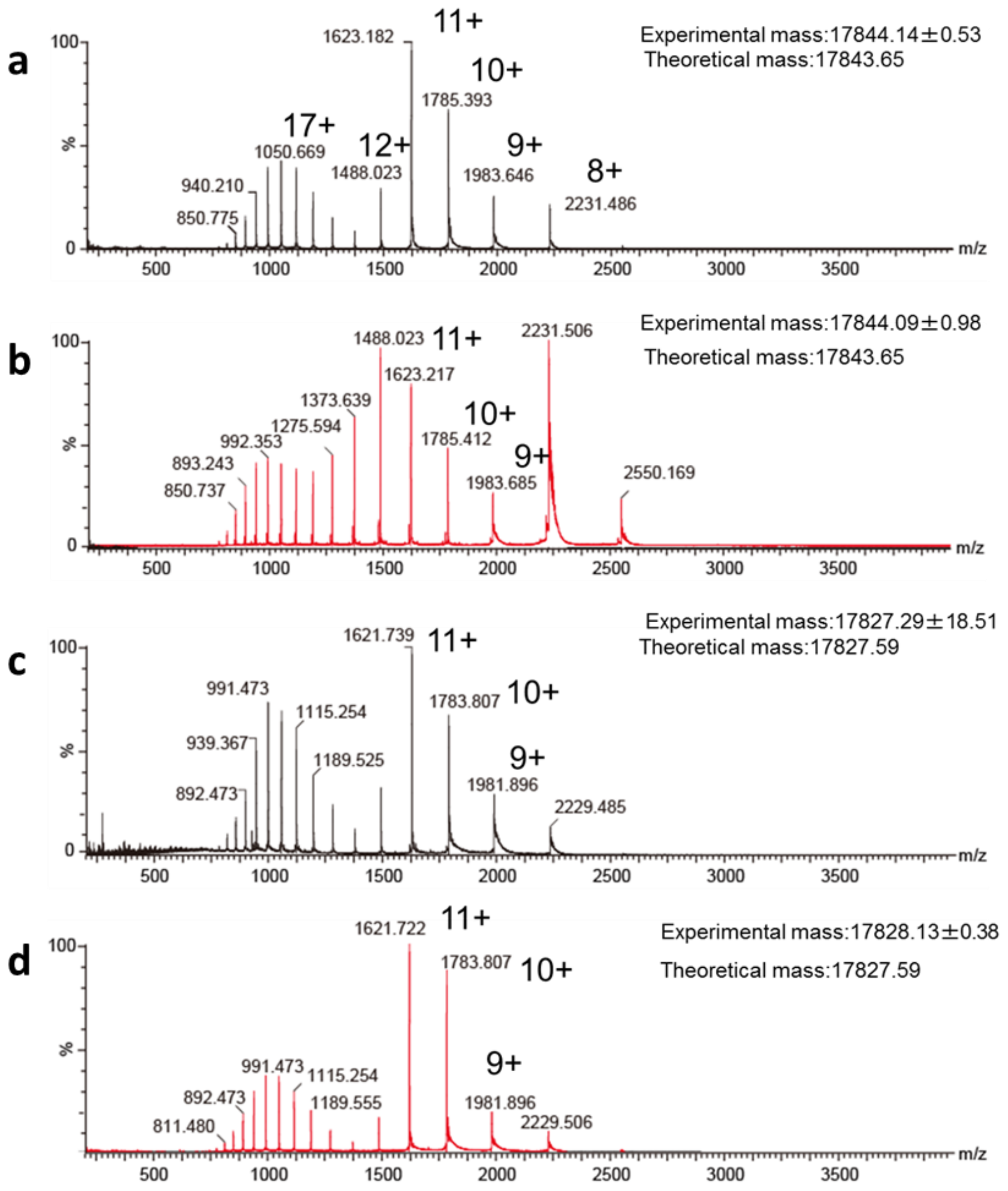

Supplementary Figure 9 ESI-MS analyses under denaturing conditions

PGRMC1 wild type (**a**: apo , **b**: haem) and C129S mutant (**c**: apo, **d**: haem) were analyzed by ESI-MS. These proteins were denatured before ESI-MS analyses by the addition of aliquots of formic acid (final concentration:30%). Data indicating experimental mass were expressed by mean±SD.

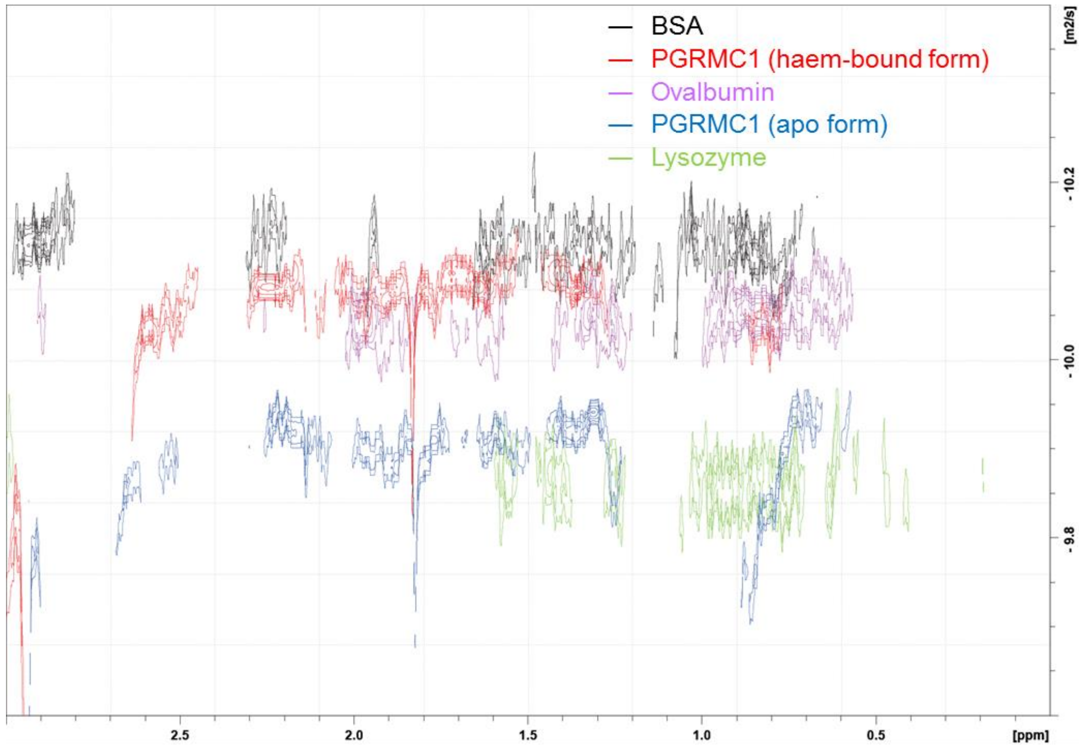

	D_{25} (DOSY) 10^{-10} [m^2 s^{-1}]	hydrodynamic radius 10^{-9} [m]	MW
BSA	0.66 – 0.70	3.50 – 3.71	66 kDa#
Ovalbumin	0.86 – 0.88	2.78 – 2.86	43 kDa#
Lysozyme	1.21 – 1.28	1.91 – 2.02	14 kDa#
PGRMC1 (apo form)	1.14 – 1.20	2.04 – 2.15*	20 kDa*
PGRMC1 (haem-bound form)	0.81 – 0.83	2.94 – 3.02*	42 kDa*

Supplementary Figure 10 DOSY analyses of apo- or haem-bound PGRMC1

Hydrodynamic radii (*r*) of PGRMC1 wt (a.a.44-195) treated with or without haemin were estimated by using the Stokes–Einstein equation with measured diffusion coefficients at 25°C, $D_{25,}$ (blue and red lines) for aliphatic region signals by DOSY measurements. The *r* values of the reference proteins (BSA (black), ovalbumin (purple) and lysozyme (green)) were obtained by the same procedure. The molecular weights (MWs) of PGRMC1 wt (a.a.44-195) treated with or without haemin were estimated using a relationship between *r* and MWs, which was derived from the linear-fitting of measured *r* values for the reference proteins with known MWs. Asterisks (*) show the data shown in Table 1. Sharps (#) show the theoretical MW of reference proteins.

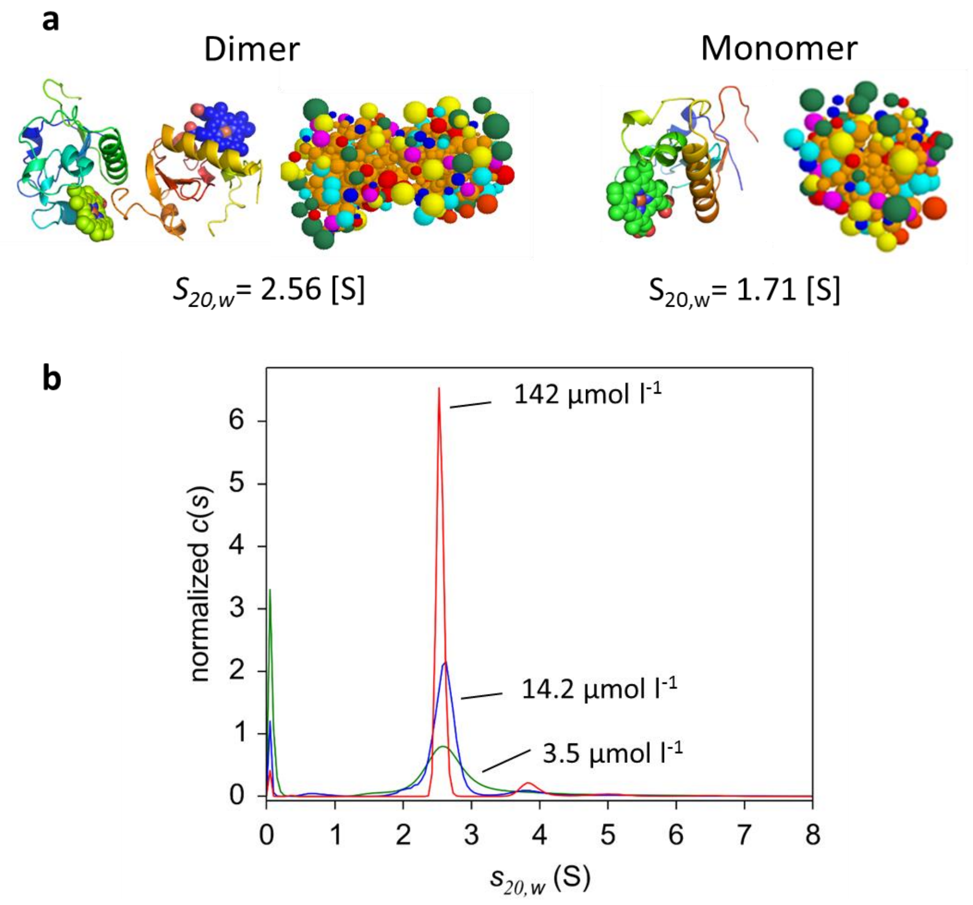

Supplementary Figure 11 PGRMC1 exists as a dimer in the presence of haem

(**a**) Sedimentation coefficients of monomer and dimer in the presence of haem were calculated using Solution Modeler (SOMO) program using PGRMC1 PDB data (Accession No.; 4X8Y). (**b**) SV-AUC experiments were performed with 142, 14.2 and 3.5 μmol l^{-1} of PGRMC1 protein (a.a.72-195) and no apparent concentration dependence such as dissociation into monomer was observed. Regarding the possible presence of higher oligomers, a weak peak at a larger size versus the dimer was observed only at the highest concentration of PGRCM1 (142 μmol l^{-1}), but not at lower concentrations. At concentration at 14.2 and 3.5 μmol l^{-1}, the single major peak was observed at sedimentation coefficient $S_{20,w}$ of ~2.5 S, indicating the detection of the dimer.

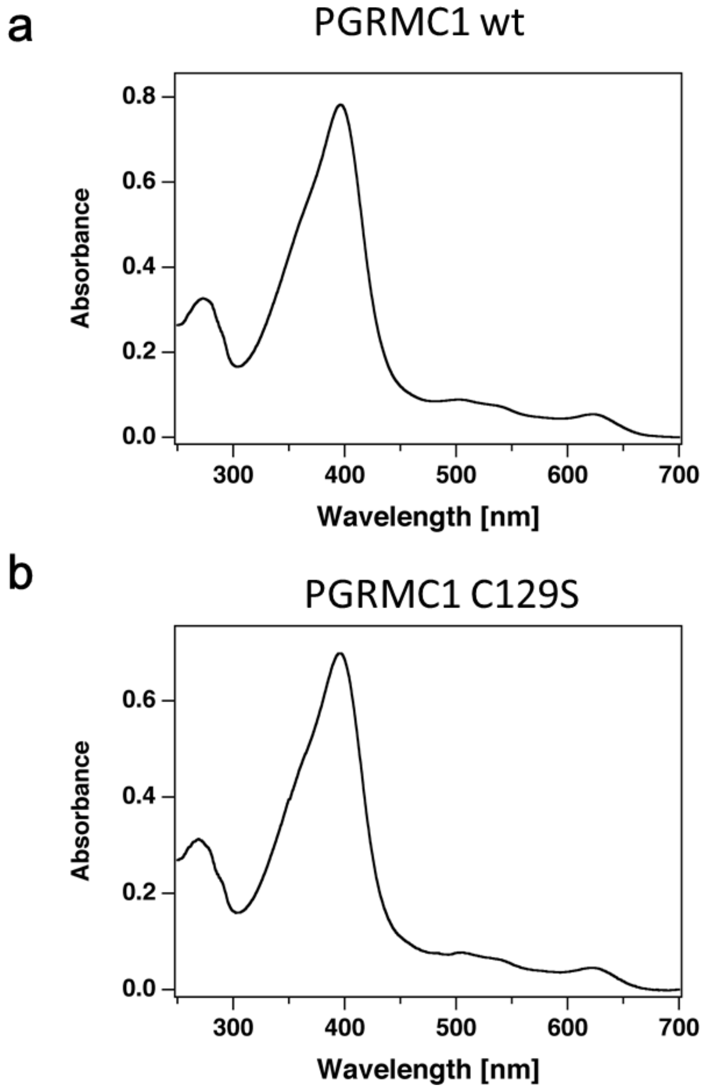

Supplementary Figure 12 UV-visible spectra of PGRMC1 wt and C129S mutant
UV-visible spectra of haem-bound PGRMC1 (a.a.44-195) wild-type (wt) (**a**) or C129S mutant (**b**), covering wavelengths in a range between 250 nm and 700 nm.

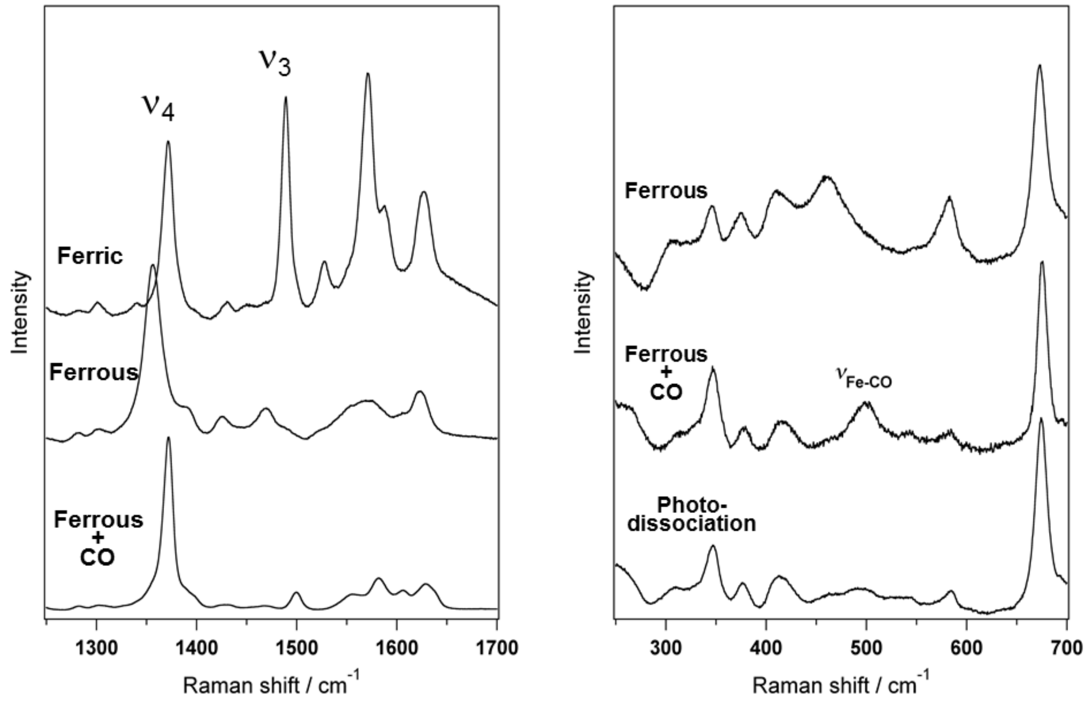

Supplementary Figure 13 **Resonance Raman spectrometry of PGRMC1**

Resonance Raman spectra of the haem–PGRMC1 (a.a.44-195) in the high-frequency (left) and low-frequency (right) regions. The spectra of the ferric, ferrous and CO-bound forms are shown. Excitation wavelength is 413.1 nm for the ferric and ferrous-CO forms and 441.6 nm for the ferrous form. As shown in the right panel, addition of CO to the ferrous form leads to the appearance of a new band at 500 cm^{-1}. This band is assignable to $\nu_{Fe\text{-}CO}$ because it disappears in response to photodissociation triggered by excitation light.

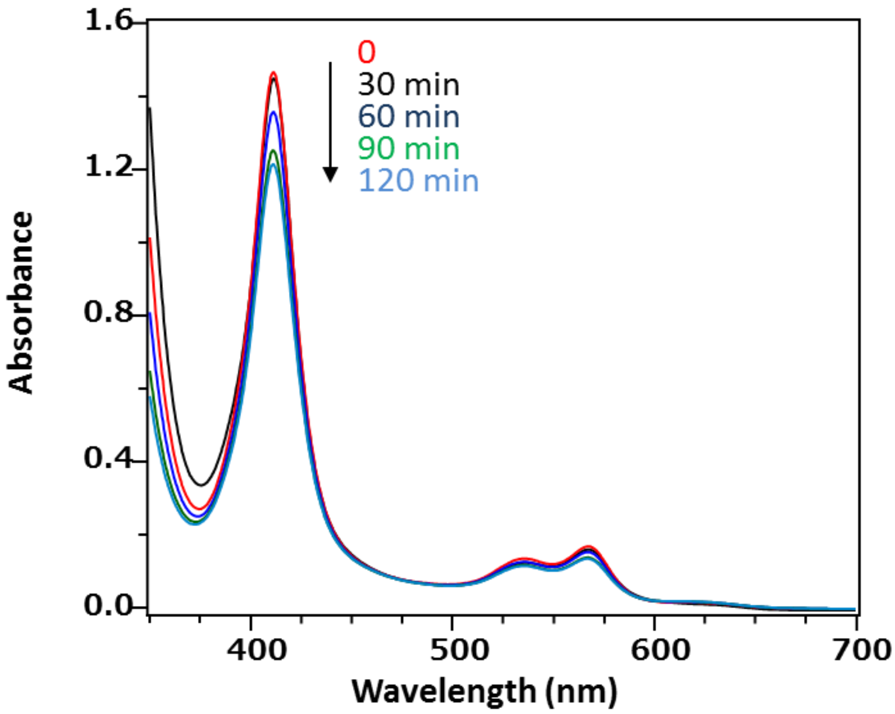

Supplementary Figure 14 Stability of the CO-bound haem of PGRMC1 under the aerobic condition

CO-bound haem of PGRMC1 (a.a.44-195) in a quartz cuvette was sealed with a rubber cap to be anaerobically prepared by addition of sodium dithionite (final concentration of 5 mmol l^{-1}) in the presence of CO gas. After the absorption spectrum was measured (0 min), the rubber cap was removed and the cuvette was placed aerobically. Absorption spectra were measured 30, 60, 90, and 120 min under aerobic conditions.

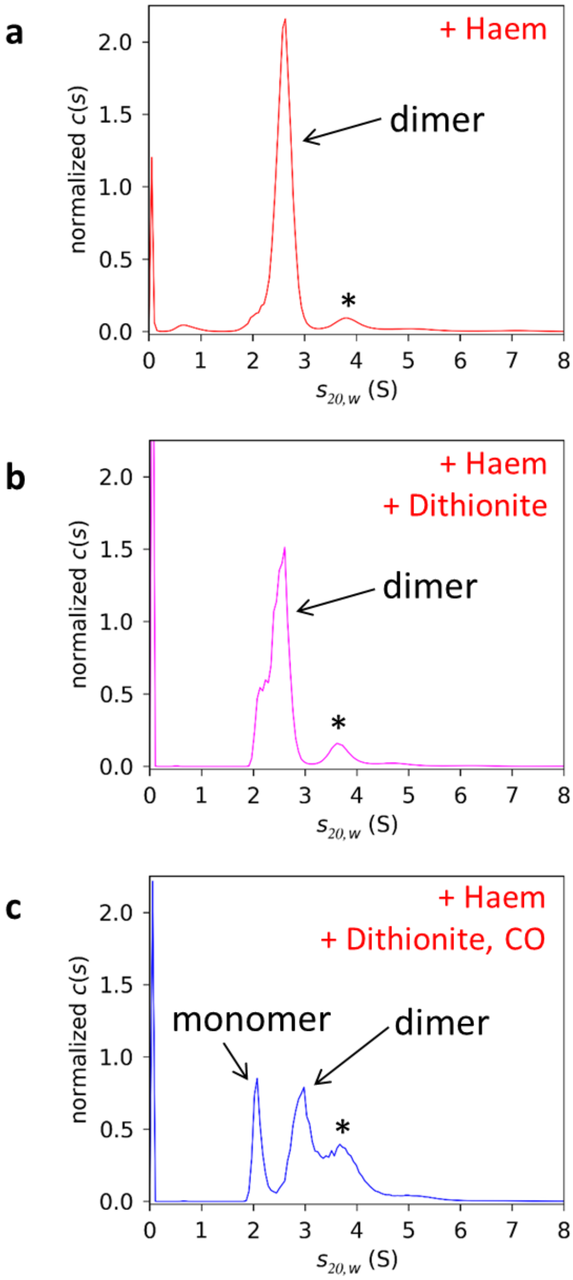

Supplementary Figure 15 Inhibitory effects of CO on haem-mediated PGRMC1 dimerization

The haem-bound PGRMC1 protein (a.a.72-195) was analysed by SV-AUC under ferric conditions (**a**), ferrous conditions by adding 5 mmol l^{-1} dithionite (**b**), or in the presence of saturated CO under the ferrous conditions (**c**). The spectrum in (**a**) is identical to that shown in Supplementary Fig. 11 at a protein concentration of 142 μmol l^{-1}. Asterisk (*) shows unidentified oligomers which were observed only at the highest concentration of PGRMC1, as shown in Supplementary Fig. 11.

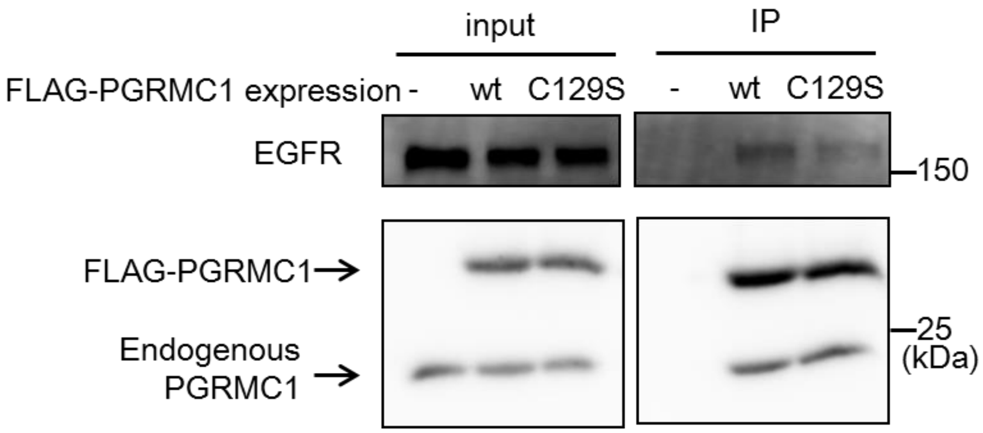

Supplementary Figure 16 PGRMC1 C129S mutant forms dimer in HCT116 cells

FLAG-PGRMC1 wild-type (wt) or C129S (full length) was over-expressed in HCT116 cells and immunoprecipitated with anti-FLAG antibody-conjugated beads. Co-immunoprecipitated proteins were detected by Western blotting.

a

PGRMC1-KD targeting sequence

229: 5'-GCATCTTCCTGCTCTACAAGA-3'

379: 5'-GCATACTCATGGCCATCAACG-3'

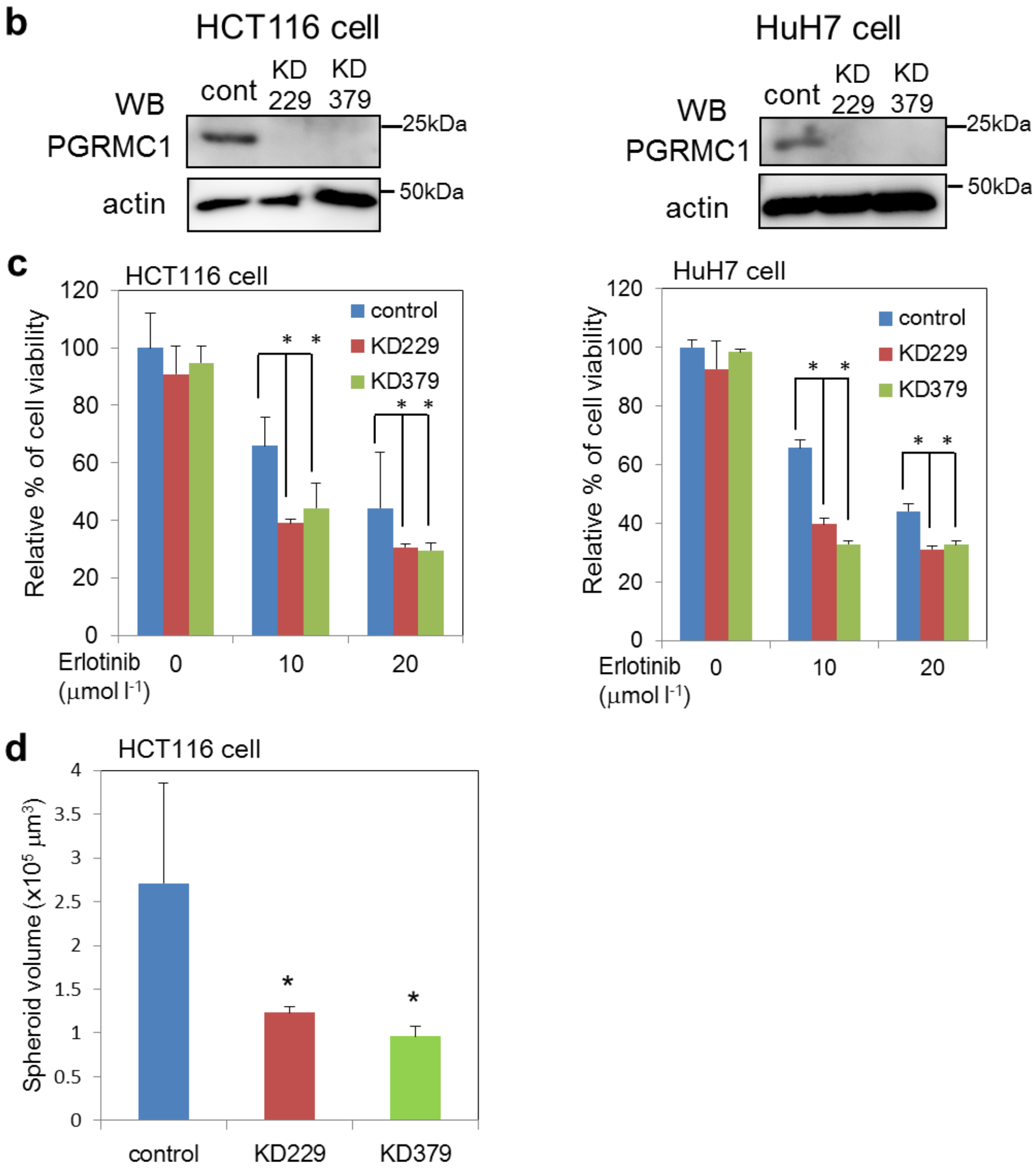

Supplementary Figure 17 Effects of PGRCM1 knockdown on cancer cell proliferation and chemoresistance

(**a**) Representative sequences of PGRMC1 knockdown shRNA introduced into lentivirus vector (upper panel). (**b**) Control and two independent shRNAs for PGRMC1 (KD229 and KD379) expressing lentivirus were infected into HCT116 cells and HuH7 cells. Cell lysates were analyzed by Western blotting. (**c**) Control and PGRMC1-KD cells were incubated with indicated amount of erlotinib for 24 h, and cell viability was analyzed by MTT assay. Data represent mean ± SD of 4 separate experiments. $^{*}P<0.01$ versus controls as judged by ANOVA with Fischer's LSD test. (**d**) Differences in relative percentages of spheroid volume among HCT116 cells treated with control shRNA and those with two-independent shPGRMC1 (KD229 and KD379). $^{*}P<0.05$ versus controls as judged by ANOVA with Fischer's LSD test.

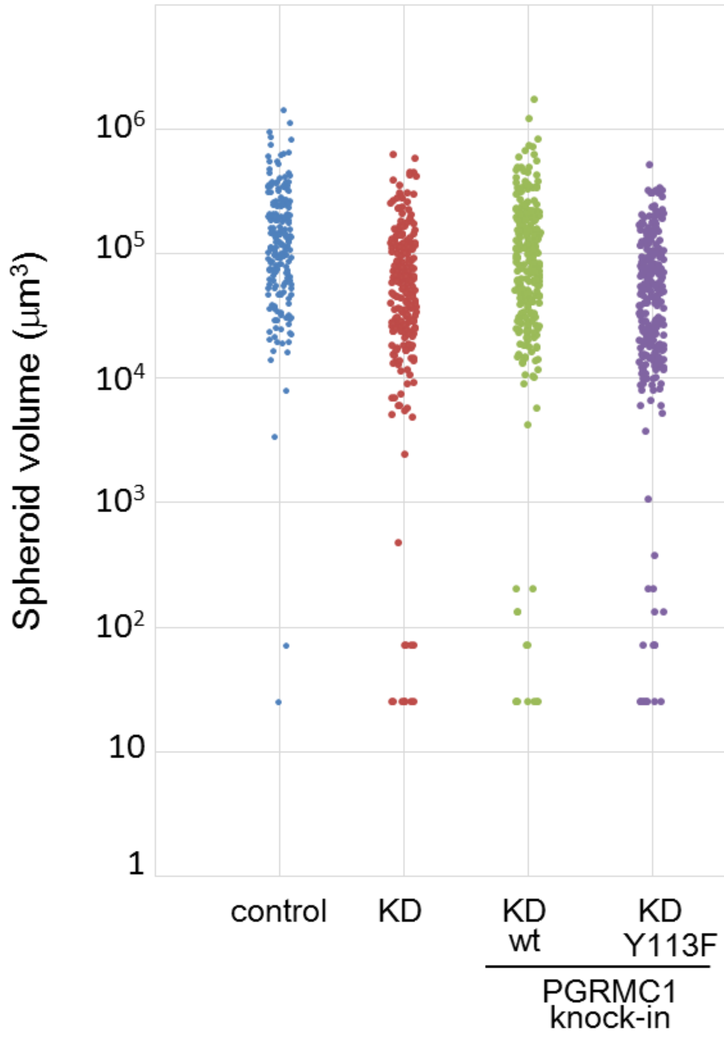

Supplementary Figure 18

Scatter plots of the difference in spheroid sizes corresponding to Figure 5c

a

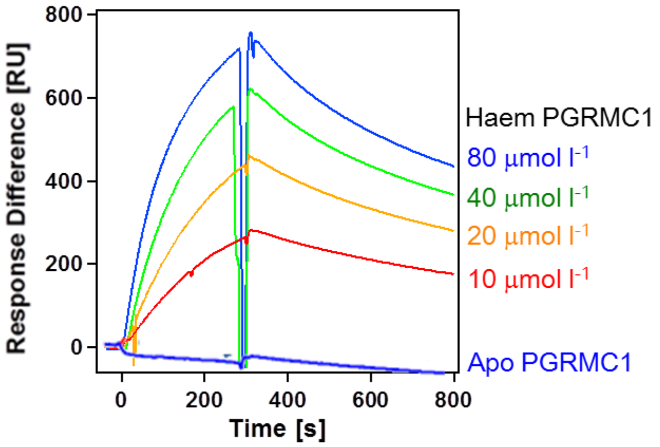

b

Protein Complex	Function	*k*a ((mol l^{-1})$^{-1}$ s^{-1})	*k*d ((mol l^{-1})$^{-1}$ s^{-1})	*K*d (mol l^{-1})
CYP51/PGRMC1	Sterol Biosynthesis	2.0×10^{2}	1.2×10^{-3}	5.9×10^{-6}
CPR/HO1	Haem Degradation	1.6×10^{3}	4.0×10^{-3}	2.4×10^{-6}
Neuroglobin/Ga_{i1}	Signal transduction	5.0×10^{2}	3.0×10^{-4}	6.0×10^{-7}

Supplementary Figure 19

Surface plasmon resonance analyses showing interactions between CYP51 and PGMRC1

(**a**) Indicated amounts of PGRMC1 in the presence or absence of haem were injected onto the CYP51-immobilized sensor surface to calculate binding affinity. (**b**) Comparison of binding affinities of CYP51/PGRMC1, CPR/HO1 and neuroglobin/Gαi1.

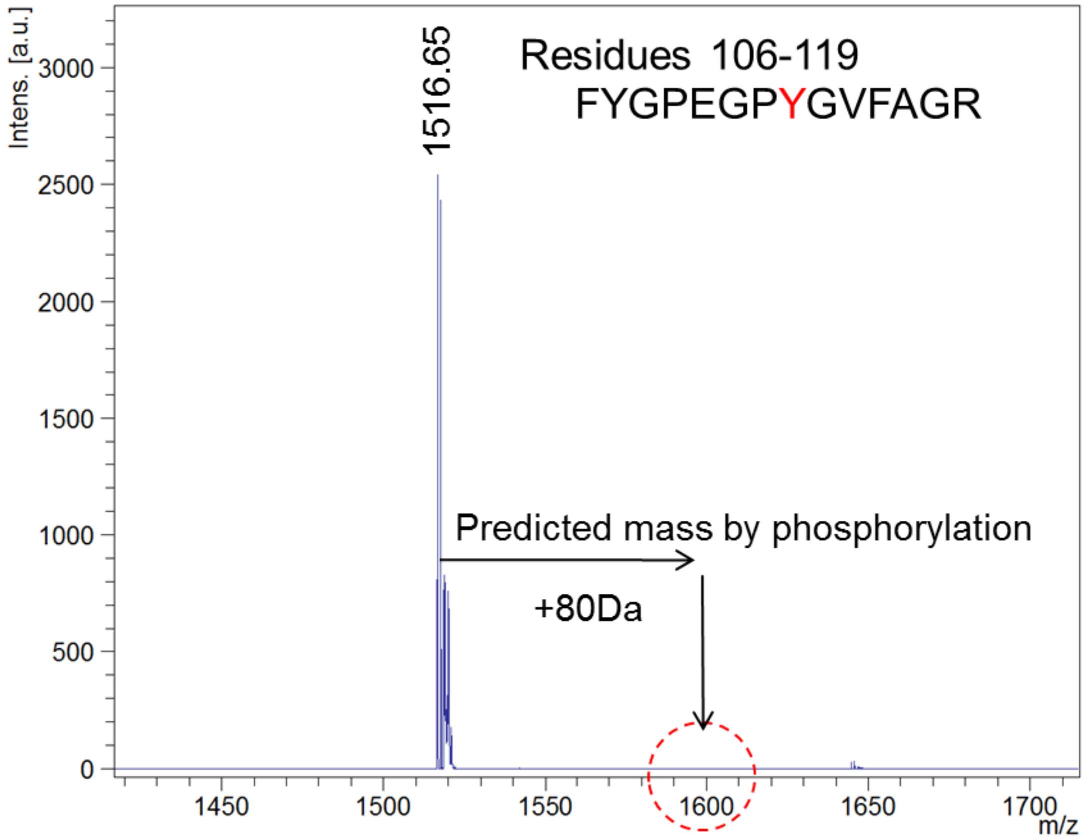

Supplementary Figure 20 ESI-MS analysis of PGMRC1 Y113 residue

FLAG-PGRMC1 (wild-type and full length) was over-expressed in HCT116 cells and immunoprecipitated with an anti-FLAG antibody-conjugated beads. The immunoprecipitants were subjected into SDS-PAGE and stained with CBB. The FLAG-PGRMC1 band was digested with trypsin to examine if the Y113 peptide fragment was phosphorylated or not (residues 106-119) by ESI-MS.

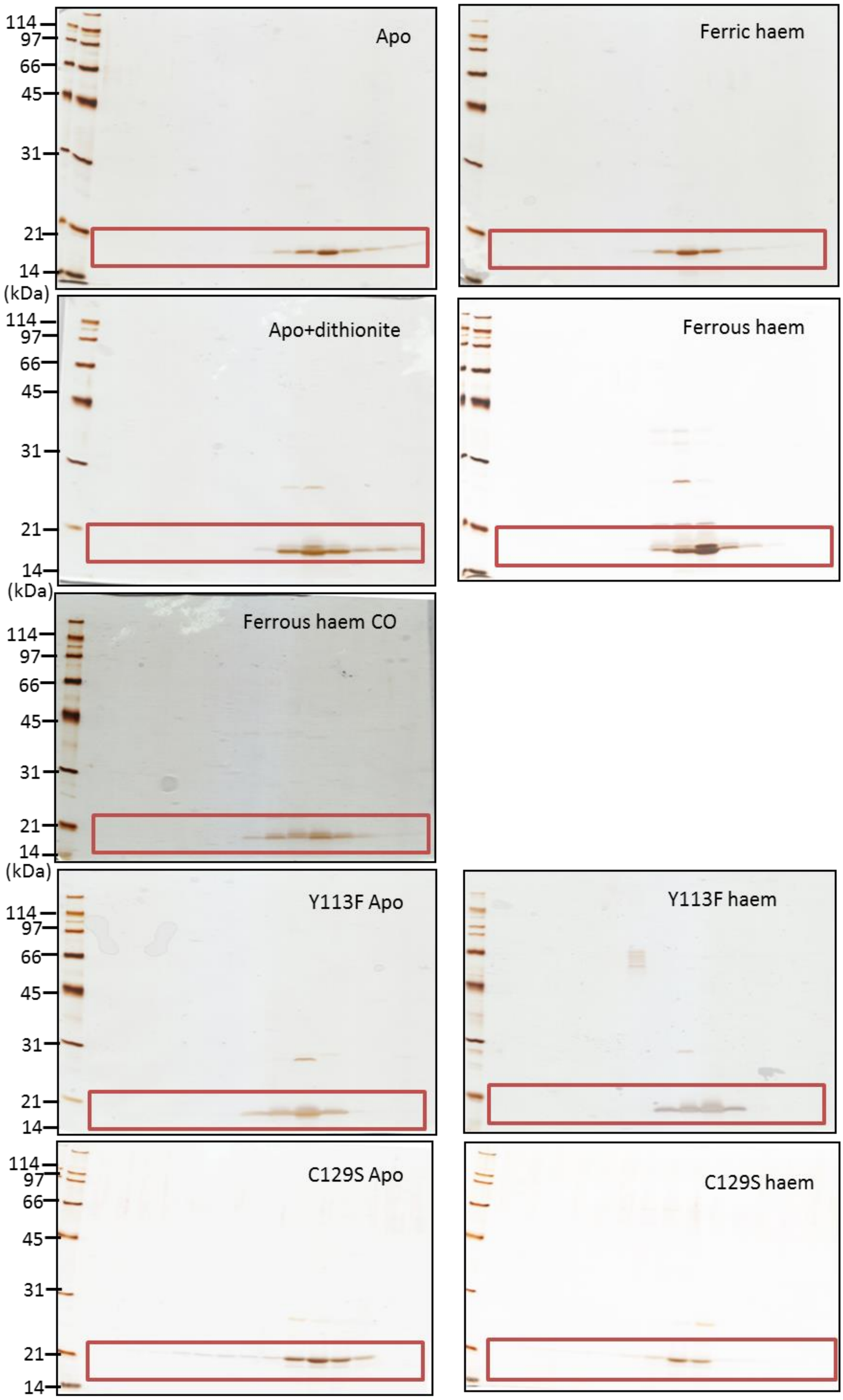

Figure 3c Full images

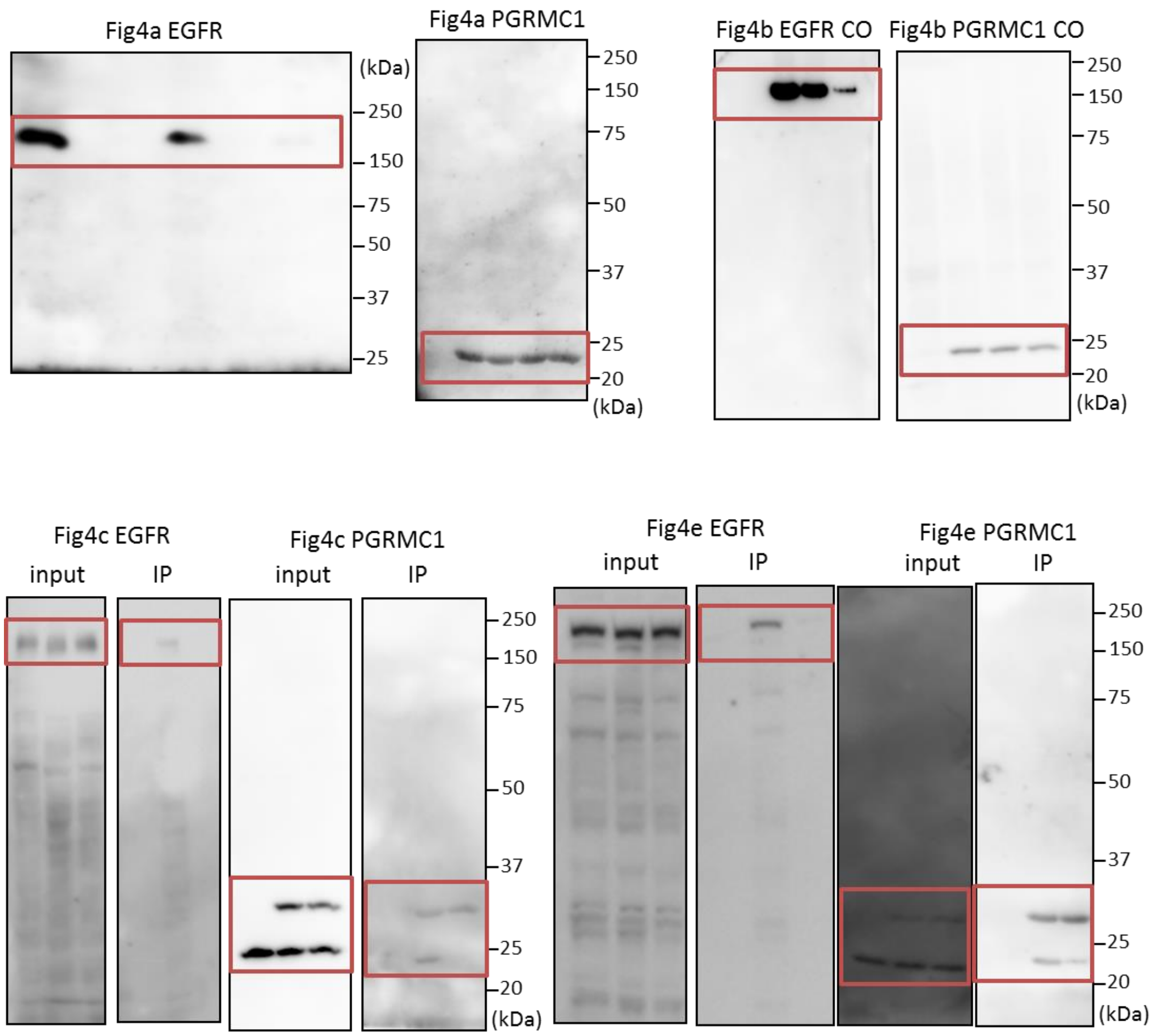

Figure 4a, b, c, e Full images

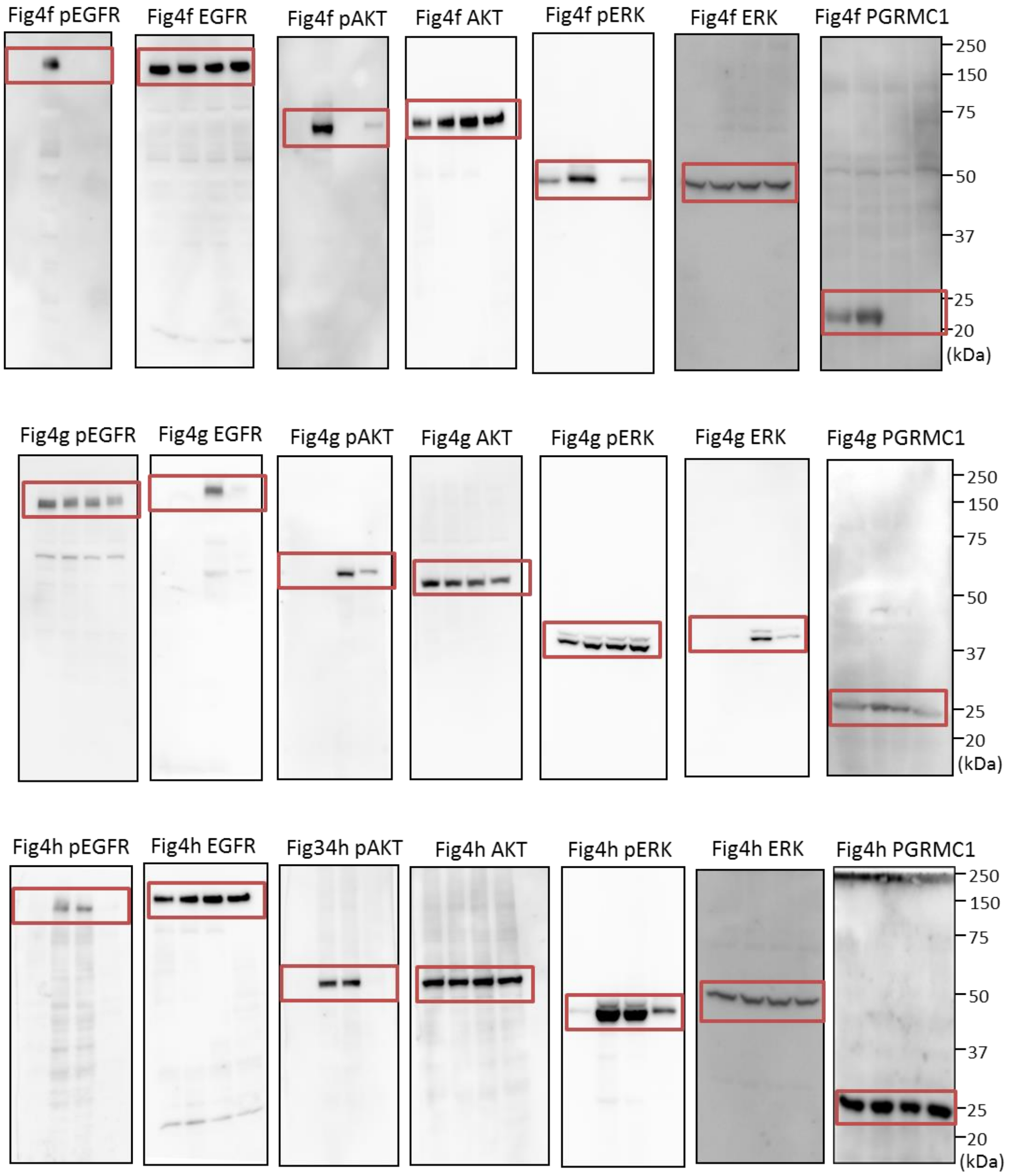

Figure 4f, g, h Full images

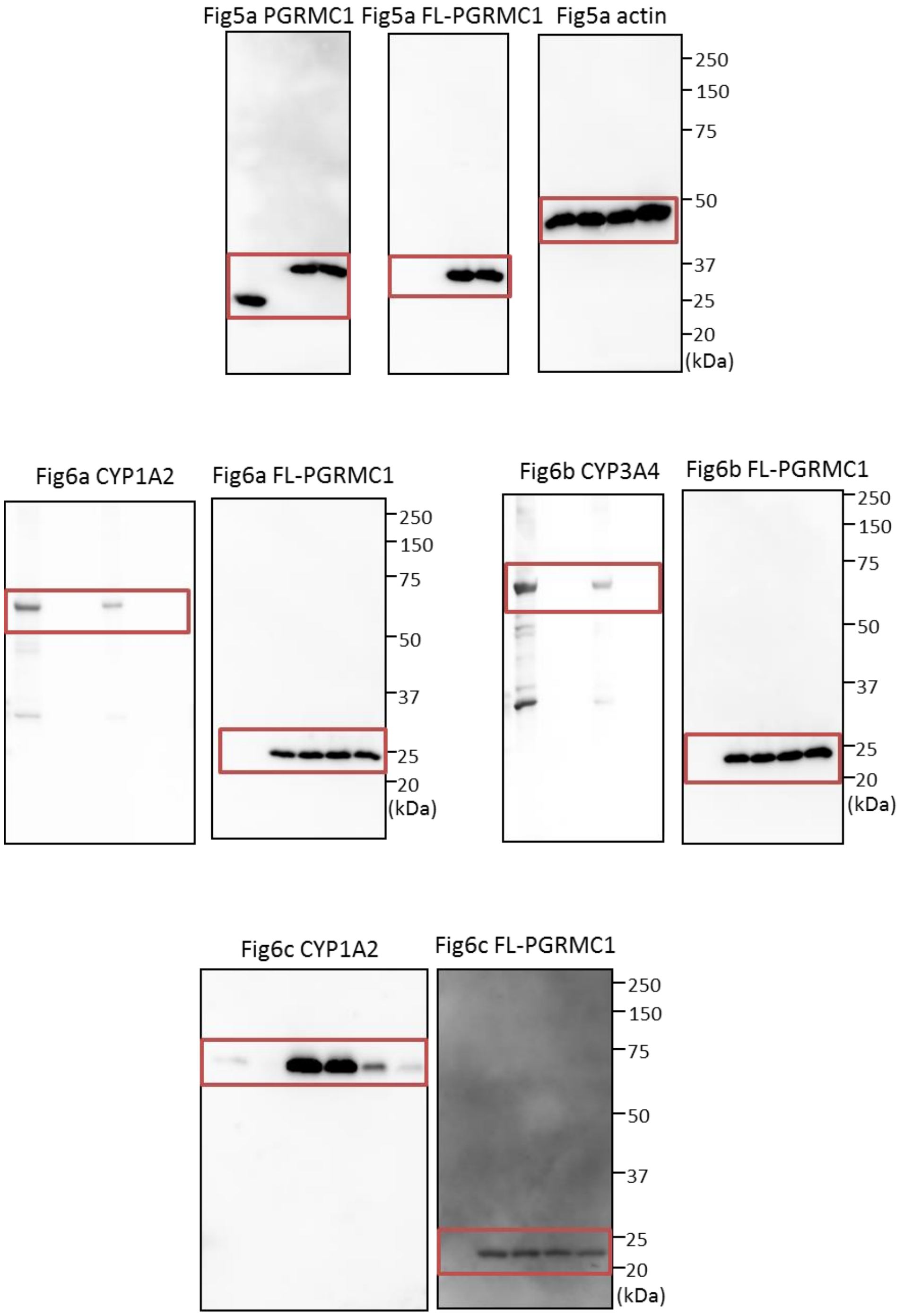

Figure5a, 6a, b, c Full images

Supplementary Figure 21 Full images of silver staining gels and Western blots

Protein	Function	K_d (nmol l^{-1})
PGRMC1	Sterol regulation	5.0 × 10
Myoglobin	Oxygen storage	1.3×10^{-5}
IRP2	Iron regulation	9.0 × 10

Supplementary Table 1 Comparison of haem-binding affinities among PGRMC1, myoglobin and IRP2

Supplementary Methods

Affinity purification of PGRMC1 by using haem-conjugated affinity nano-beads

Control, PP-IX and haem-fixed affinity nano-beads were prepared as previously described[1, 2]. Briefly, PP-IX or haemin was incubated at 1 mmol l^{-1} with equal amounts of *N*-hydroxysuccinimide and 1-ethyl-3-(3-dimethylaminopropyl) carbodiimide (Dojindo) for 2 h at room temperature and then reacted overnight with amino-modified affinity beads. For purification of haem-binding proteins, 0.2 mg of beads were equilibrated with the binding buffer (20 mmol l^{-1} HEPES (pH7.9), 100 mmol l^{-1} NaCl, 1 mmol l^{-1} $MgCl_2$, 0.2 mmol l^{-1} EDTA, 10% glycerol, 1 mmol l^{-1} DTT, 0.2 mmol l^{-1} PMSF, 0.1% NP40), and incubated with 1 mg ml^{-1} of mouse liver extracts at 4˚C for 1 h. Bound proteins were eluted with SDS-loading dye, analysed by SDS-PAGE and then visualized by silver staining (Wako). Bound proteins were subjected to in-gel digestion by trypsin, and the fragments of the peptides were analysed by ESI-MS (Hitachi, NanoFrontier).

Primers for plasmid constructions

PGRMC1 full-length forward:

5’-TTTGGATCCATGGCTGCCGAGGATGTGGTGGCG-3’

PGRMC1 stop reverse:

5’-TTTGTCGACTTCATCATTTTTCCGGGCACTCTC-3’

PGRMC1 Δ43 forward:

5’-TTTGAATTCAAGATCGTGCGCGGGGACCAG-3’

PGRMC1 Δ71 forward:

5’-TTTGAATTCGACTTCACCCCCGCCGAGCTG-3’

PGRMC1 Δ43 FXa site forward:

5’-TTTTGGATCCATCGAAGGTCGTGAATTCAAGATCGTGCGCGGGGACCAG-3’

PGRMC1 C-FLAG reverse:

5’-TTTTGTCGACTTACTTGTCGTCATCGTCTTTGTAGTCTTCATCATTTTTCCGGGCACTCTC-3’

PGRMC1 shRNA229 sense:

5’-GCATCTTCCTGCTCTACAAGATTCAAGAGATCTTGTAGAGCAGGAAGATGCTTTT-3’

PGRMC1 shRNA229 antisense:

5’-GATCAAAAAGCATCTTCCTGCTCTACAAGATCTCTTGAATCTTGTAGAGCAGGAAGATGCCA-3’

PGRMC1 shRNA379 sense:

5’-GCATACTCATGGCCATCAACGTTCAAGAGACGTTGATGGCCATGAGTATGCTTTTT-3’

PGRMC1 shRNA379 antisense:

5’-GATCAAAAAGCATACTCATGGCCATCAACGTCTCTTGAACGTTGATGGCCATGAGTATGCCA-3’

PGRMC1 Y113F forward:

5’-CGAGGGGTTTGGGGTCTTTGCTG-3’

PGRMC1 Y113F reverse:

5’-CAGCAAAGACCCCAAACGGCCCCTCG-3’

PGRMC1 C129S forward:

5’-GGCTTTGCCACATTTAGCCTGGATAAGGAAGCAC-3’

PGRMC1 C129S reverse:

5’-GTGCTTCCTTATCCAGGCTAAATGTGGCAAGGCC-3’

PGRMC1 shRNA resistant forward:

5’-GGCCTCTGCATATTTCTCCTGTATAAGATCGTGCGC-3’

PGRMC1 shRNA resistant reverse:

5’-GCGCACGATCTTATACAGGAGAAATATGCAGAGGCC-3’

Preparation of recombinant proteins

Human CYP51, in which the N-terminal transmembrane anchor domain (residues 1-59) was replaced with the MAKKT sequence to prepare the soluble enzyme[3], was expressed in HMS174 (Novagen). The enzyme was purified using a HiTrap column (GE Healthcare) with 50 mmol l^{-1} phosphate buffer (pH7.4) containing 20% glycerol, followed by a gel-filtration column (HiLoad 16/60 Superdex 200; GE Healthcare)[4].

NMR Spectroscopy

Apo- and haem-bound PGRMC1 (500 μmol l^{-1}) in 50 mmol l^{-1} phosphate buffer (pH7.0) containing 5% D_2O were used for the NMR measurements. Deuterated samples were used for NMR analysis of the heam-bound form of PGRMC1. NMR spectra were measured at 25°C using a Bruker Avance DRX 600 spectrometer equipped with a triple resonance ($^1H/^{13}C/^{15}N$) cryoprobe. The ^1H-^{15}N HSQC, HNCO, HN(CA)CO, HNCA, HN(CO)CA, CBCA(CO)NH and HNCACB spectra were measured for sequential assignments of the backbone ^1H, ^{13}C and ^{15}N chemical shifts of the apo form. ^1H-^{15}N transverse relaxation-optimized spectroscopy (TROSY) types of 2D and 3D NMR measurements were performed for the haem-bound form of PGRMC1. NMR data were processed using the program NMRDraw[5], and signal assignments were performed using the programs Kujira[6] and NMRView[7]. The prediction of secondary structure of PGRMC1 in solution was estimated by the program TALOS+[8].

Chemical shift assignments of the apo and haem-bound forms were compared after adjusting 1/2 J_{HN} on both the ^1H and ^{15}N axes of the TROSY spectra. The weighted chemical shift changes of the backbone amide proton (HN) and nitrogen (N) atoms were calculated according to the following equation: $\Delta\delta = \sqrt{(\Delta NH)^2 + (\Delta N/5)^2}$.

Resonance Raman measurements

Resonance Raman spectra were obtained with a single monochromator (SPEX

500M; Jobin-Yvon) equipped with a liquid nitrogen-cooled CCD detector (Spec-10:400B/LN; Roper Scientific). The excitation wavelength used was 413.1 nm from a krypton ion laser (BeamLok 2060; Spectra Physics). To prevent photodissociation, laser power at the sample point was adjusted to approximately 5mW for the oxidized and dithionite-reduced forms and to 0.1 mW for the CO-bound form. Raman shifts were calibrated with indene, CCl_4, acetone and an aqueous solution of ferrocyanide. The accuracy of the peak positions of well-defined Raman bands was ±1 cm^{-1}. The sample concentrations for the RR experiments were about 10 μmol l^{-1} in 50 mmol l^{-1} Tris-HCl (pH8.0), 300 mmol l^{-1} NaCl.

Surface plasmon resonance analyses

Kinetic evaluation of PGRMC1 binding to human CYP51 was carried out on a Biacore J system (GE Healthcare). The NTA sensor chip was activated with an injection of 500 μmol l^{-1} $NiCl_2$ (pH 7.4); then, human CYP51 (in 50 mmol l^{-1} Tris-HCl (pH7.0)) was immobilized on a NTA sensor chip at 25 °C. Each PGRMC1 protein (10–80 μmol l^{-1}) in HBS-P buffer (0.01 mol l^{-1} HEPES, 0.15 mol l^{-1} NaCl, 0.005% Surfactant P20, pH 7.4) was injected at a constant flow rate of 20 μl per min for 300 sec. The kinetic constants were calculated using BIA evaluation software, version 4.1, assuming a 1:1 binding model (GE Healthcare).

Supplementary References

1. Azuma, M. *et al.* Adenine nucleotide translocator transports haem precursors into mitochondria. *PloS one* **3**, e3070 (2008).
2. Kabe, Y. *et al.* Porphyrin accumulation in mitochondria is mediated by 2-oxoglutarate carrier. *The Journal of biological chemistry* **281**, 31729-31735 (2006).
3. von Wachenfeldt, C., Richardson, T.H., Cosme, J. & Johnson, E.F. Microsomal P450 2C3 is expressed as a soluble dimer in Escherichia coli following modification of its N-terminus. *Archives of biochemistry and biophysics* **339**, 107-114 (1997).
4. Matsuura, K. *et al.* Dioxygen reduction by bo-type quinol oxidase from Escherichia coli studied by submillisecond-resolved freeze-quench EPR spectroscopy. *Biochemistry* **43**, 2288-2296 (2004).
5. Delaglio, F. *et al.* NMRPipe: a multidimensional spectral processing system based on UNIX pipes. *Journal of biomolecular NMR* **6**, 277-293 (1995).
6. Kobayashi, N. *et al.* KUJIRA, a package of integrated modules for systematic and interactive analysis of NMR data directed to high-throughput NMR structure studies. *Journal of biomolecular NMR* **39**, 31-52 (2007).
7. Johnson, B.A. & Blevins, R.A. NMR View: A computer program for the visualization and analysis of NMR data. *Journal of biomolecular NMR* **4**, 603-614 (1994).
8. Shen, Y., Delaglio, F., Cornilescu, G. & Bax, A. TALOS+: a hybrid method for predicting protein backbone torsion angles from NMR chemical shifts. *Journal of biomolecular NMR* **44**, 213-223 (2009).
